# Supplementary figures and images for: Maternal knowledge, attitudes and practices related to neonatal jaundice and associated factors in Shenzhen, China: a facility-based cross-sectional study
Source: BMJ Open. 2022 Aug 24;12(8):e057981. doi: 10.1136/bmjopen-2021-057981 (PMC9413169; doi:10.1136/bmjopen-2021-057981)

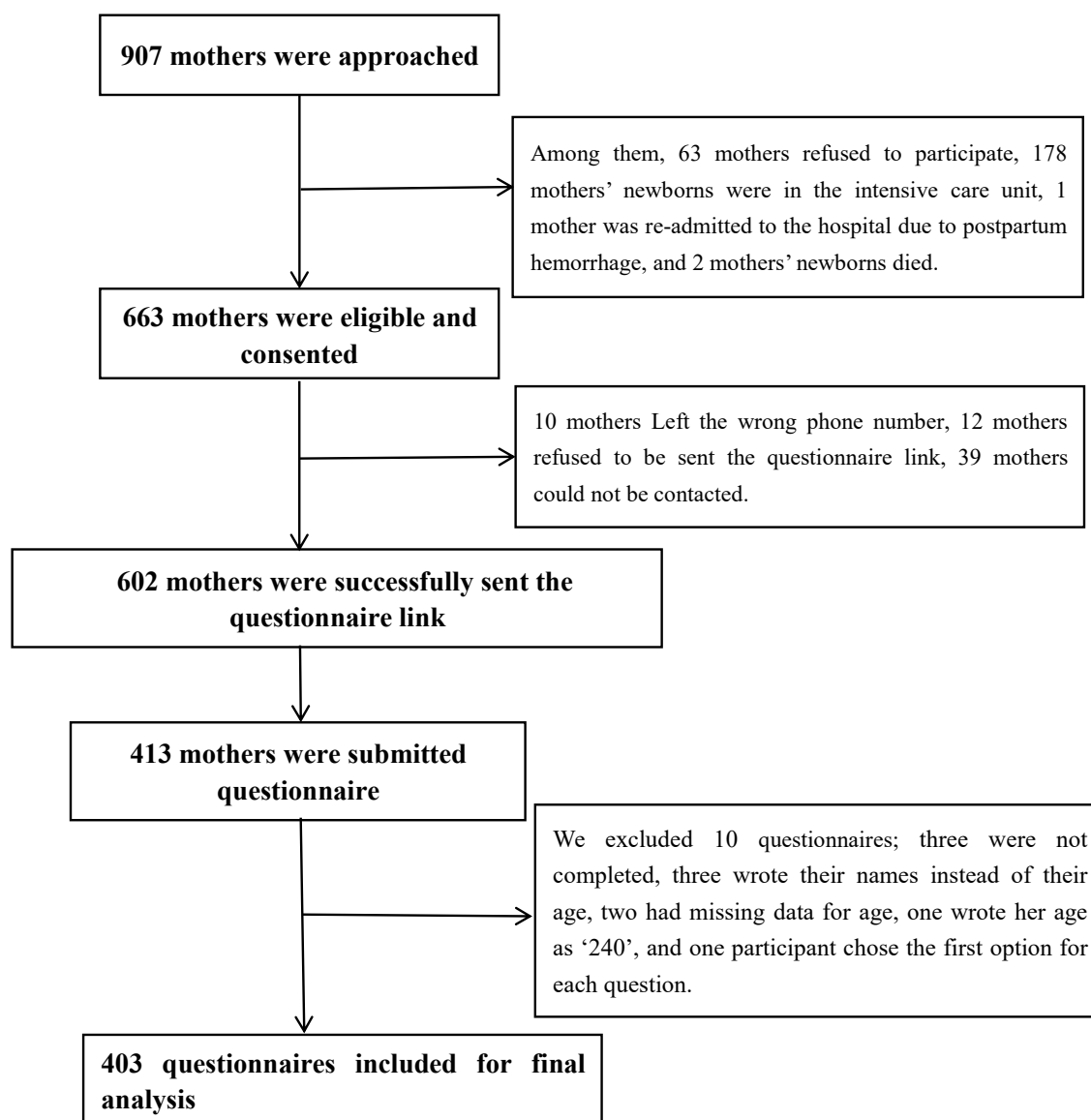

Supplement: Supplementary data [file bmjopen-2021-057981supp001.pdf]
