# Supplementary material for: Maternal knowledge, attitudes and practices related to neonatal jaundice and associated factors in Shenzhen, China: a facility-based cross-sectional study
Source: BMJ Open. 2022 Aug 24;12(8):e057981. doi: 10.1136/bmjopen-2021-057981 (PMC9413169; doi:10.1136/bmjopen-2021-057981)
Supplement: Supplementary data [file bmjopen-2021-057981supp002.pdf]

Supplementary table 1. Chi-square analyses of maternal knowledge, attitudes and practices related to neonatal jaundice (N=403)

Variables		Knowledge*		Attitude*		Behaviour*	
		χ^2	*p*	χ^2	*p*	χ^2	*p*
**Sociodemographic data**							
Age(years)	19~27<br>28~32<br>33~45	2.008	0.366	1.385	0.500	0.292	0.864
Blood group	O<br>A<br>B<br>AB	1.668	0.644	3.873	0.276	4.244	0.236
Education level	High school and below<br>University<br>Postgraduate and above	**16.921**	**0.000**	**9.800**	**0.007**	**6.084**	**0.048**
Occupation	Employed<br>Self-employed<br>Housewife<br>Others	**15.279**	**0.002**	2.007	0.571	6.865	0.076
Average family monthly income (RMB)	≤5000<br>5001~10000<br>10001~20000<br>20001~30000<br>≥30001	**10.444**	**0.034**	6.849	0.144	8.683	0.070
Time from the place of residence to the delivery hospital (minutes)	≤10<br>10~30<br>30~60<br>≥60	1.458	0.692	3.289	0.349	1.635	0.652
**Delivery history**							
Parity	Primiparous<br>Multipara	0.021	0.885	**4.536**	**0.033**	0.629	0.428
Delivery mode	Spontaneous vaginal<br>Caesarean section	0.014	0.907	0.152	0.696	0.500	0.479
**Infant's Information**							
Sex	Male<br>Female	**6.409**	**0.011**	2.241	0.134	0.804	0.370

Birth weight[1]	Low<br>Normal<br>Hight	1.008	0.615	2.034	0.402	4.216	0.130
Feeding way	Exclusive breastfeeding<br>Mixed feeding<br>Exclusive Formula-feeding	5.479	0.065	0.873	0.647	5.604	0.062
Cranial hematoma[2]	Yes<br>No<br>Not sure	**7.288**	**0.026**	0.342	0.843	0.380	0.827
Whether to pass meconium with in 24 hours	Yes<br>No	0.052	0.820	1.626	0.202	3.247	0.072
Predischarge bilirubin level	Normal<br>Hight	1.253	0.263	0.022	0.882	1.837	0.175
"yuesao" [3]	Yes<br>No	1.067	0.320	**11.878**	**0.001**	0.327	0.567
**Previous experience/exposure to NNJ**							
Prior health education on neonatal jaundice	Yes<br>No	**8.824**	**0.003**	0.179	0.672	**9.236**	**0.002**
Previous child history of neonataljaundice	Yes<br>No	1.067	0.302	0.140	0.708	0.133	0.715
Current child admitted to the hospital for treatment due to jaundice after disch arge	Yes<br>No	0.358	0.549	0.012	0.912	3.156	0.076
Family history/friends with NNJ history（N=373)	Yes<br>No	0.351	0.554	0.100	0.752	0.860	0.354
Mother’s knowledge level	good<br>poor	*NA*		**9.478**	**0.002**	**35.751**	**0.000**
Mother’s attitude level	good<br>poor	*NA*		*NA*		**7.179**	**0.007**

Note: *NA*, not applicable. *Knowledge attitude and behaviour were all divided into two levels (po or or good)
