# Supplementary material for: Maternal knowledge, attitudes and practices related to neonatal jaundice and associated factors in Shenzhen, China: a facility-based cross-sectional study
Source: BMJ Open. 2022 Aug 24;12(8):e057981. doi: 10.1136/bmjopen-2021-057981 (PMC9413169; doi:10.1136/bmjopen-2021-057981)
Supplement: Supplementary data [file bmjopen-2021-057981supp003.pdf]

Supplementary table 2. Binary logistic regression analysis of maternal knowledge, attitudes and practices related to neonatal jaundice for mothers with yuesao (N=138)

Variables	Classification	Knowledge			Attitude			Practices		
		OR	95%*CI*	*P*	*OR*	95%*CI*	*P*	*OR*	95%*CI*	*P*
Education level	High school and below (Ref.)			**0.048**			**0.033**		*NI*	
	College and undergraduate course	7.123	1.387~36.588	0.019	7.683	1.583~37.297	0.011			
	Postgraduate student or above	4.316	0.645~28.877	0.132	9.900	1.539~63.689	0.016			
Average family monthly income (RMB)	≤5000 (Ref.)		*NI*			*NI*			*NI*	
	5001~10000									
	10001~20000									
	20001~30000									
	≥30001									
Prior education on neonatal jaundice	Yes (Ref.)		*NI*			*NI*			*NI*	
	No									
Neonate sex	Female (Ref.)		*NI*			*NI*			*NI*	
	Male									
Occupation	Employed (Ref.)			**0.017**					*NI*	
	Self-employed	0.131	0.034~0.496	0.003		*NI*				

	Homemaker	1.182	0.393~3.559	0.766						
	Other	0.306	0.390~0.064	0.306						
Knowledge level	Poor (Ref.)		*NA*			*NI*				
	good							0.505	0.254~1.003	0.051
Attitude level	poor (Ref.)		*NI*			*NA*			*NI*	
	good									

Note: *OR,* odds ratio; *CI* confidence interval; *NI*, not included in the final logistic regression analysis; *NA*, not applicable.

Supplementary table 3. Binary logistic regression analysis of maternal knowledge, attitudes and practices related to neonatal jaundice for mothers without yuesao (N=265)

**Variables**	**Classification**	**Knowledge**			**Attitude**			**Practices**		
		OR	95%*CI*	*P*	*OR*	95%*CI*	*P*	*OR*	95%*CI*	*P*
Education level	High school and below (Ref.)			**0.001**						
	College and undergraduate course	2.832	1.430~5.610	0.030		*NI*			*NI*	
	Postgraduate student or above	20.140	3.036~133.601	0.002						
Average family monthly income (RMB)	≤5000 (Ref.)		*NI*			*NI*			*NI*	
	5001~10000									
	10001~20000									
	20001~30000									

	≥30001									
Prior education on neonatal jaundice	Yes (Ref.)					*NI*			*NI*	
	No	5.335	1.654~17.208	**0.005**						
Neonate sex	Female (Ref.)					*NI*			*NI*	
	Male	2.083	1.233~3.518	**0.006**						
Occupation	Employed (Ref.)		*NI*			*NI*			*NI*	
	Self-employed									
	Homemaker									
	Other									
Knowledge level	Poor (Ref.)		*NA*							
	good				1.734	1.059~2.847	**0.029**	3.012	1.757~5.165	**0.000**
Attitude level	poor (Ref.)		*NI*			*NA*				
	good							1.967	1.161~3.333	**0.012**

Note: *OR,* odds ratio; *CI* confidence interval; *NI*, not included in the final logistic regression analysis; *NA*, not applicable.
