# Supplementary material for: Maternal knowledge, attitudes and practices related to neonatal jaundice and associated factors in Shenzhen, China: a facility-based cross-sectional study
Source: BMJ Open. 2022 Aug 24;12(8):e057981. doi: 10.1136/bmjopen-2021-057981 (PMC9413169; doi:10.1136/bmjopen-2021-057981)
Supplement: Author's manuscript [file bmjopen-2021-057981.draft_revisions.pdf]

BMJ Open

BMJ Open is committed to open peer review. As part of this commitment we make the peer review history of every article we publish publicly available.

When an article is published we post the peer reviewers' comments and the authors' responses online. We also post the versions of the paper that were used during peer review. These are the versions that the peer review comments apply to.

The versions of the paper that follow are the versions that were submitted during the peer review process. They are not the versions of record or the final published versions. They should not be cited or distributed as the published version of this manuscript.

BMJ Open is an open access journal and the full, final, typeset and author-corrected version of record of the manuscript is available on our site with no access controls, subscription charges or pay-per-view fees (http://bmjopen.bmj.com).

If you have any questions on BMJ Open's open peer review process please email
info.bmjopen@bmj.com

BMJ Open

Maternal knowledge, attitudes and practices related to neonatal jaundice and associated factors in Shenzhen, China: a facility-based cross-sectional study.

Journal:	*BMJ Open*
Manuscript ID	bmjopen-2021-057981
Article Type:	Original research
Date Submitted by the Author:	05-Oct-2021
Complete List of Authors:	Huang, Ying; Shenzhen Hospital of Southern Medical University, Department of Nursing<br>Chen, Ling; Shenzhen Hospital of Southern Medical University, Department of Nursing<br>Wang, Xiaojiao; Shenzhen Hospital of Southern Medical University, Department of Nursing<br>Zhao, Chun; Shenzhen Hospital of Southern Medical University, Department of Nursing<br>Guo, Zonglian; Shenzhen Hospital of Southern Medical University, Department of Obstetrics<br>Li, Jue; Shenzhen Hospital of Southern Medical University, Department of Nursing<br>Yang, Fang; Shenzhen Maternity and Child Healthcare Hospital, Department of Obstetrics<br>Cai, Wenzhi; Shenzhen Hospital of Southern Medical University,
Keywords:	NEONATOLOGY, MEDICAL EDUCATION & TRAINING, Child protection < PAEDIATRICS

SCHOLARONE™
Manuscripts

I, the Submitting Author has the right to grant and does grant on behalf of all authors of the Work (as defined in the below author licence), an exclusive licence and/or a non-exclusive licence for contributions from authors who are: i) UK Crown employees; ii) where BMJ has agreed a CC-BY licence shall apply, and/or iii) in accordance with the terms applicable for US Federal Government officers or employees acting as part of their official duties; on a worldwide, perpetual, irrevocable, royalty-free basis to BMJ Publishing Group Ltd ("BMJ") its licensees and where the relevant Journal is co-owned by BMJ to the co-owners of the Journal, to publish the Work in this journal and any other BMJ products and to exploit all rights, as set out in our licence.

The Submitting Author accepts and understands that any supply made under these terms is made by BMJ to the Submitting Author unless you are acting as an employee on behalf of your employer or a postgraduate student of an affiliated institution which is paying any applicable article publishing charge ("APC") for Open Access articles. Where the Submitting Author wishes to make the Work available on an Open Access basis (and intends to pay the relevant APC), the terms of reuse of such Open Access shall be governed by a Creative Commons licence – details of these licences and which Creative Commons licence will apply to this Work are set out in our licence referred to above.

Other than as permitted in any relevant BMJ Author's Self Archiving Policies, I confirm this Work has not been accepted for publication elsewhere, is not being considered for publication elsewhere and does not duplicate material already published. I confirm all authors consent to publication of this Work and authorise the granting of this licence.

For peer review only

Title: Maternal knowledge, attitudes and practices related to neonatal jaundice and asso ciated factors in Shenzhen, China: a facility-based cross-sectional study

Basic information about the co-authors:

Ying Huang Southern Medical University; School of Nursing; Guangzhou; China

Ling Chen Shenzhen Hospital, Southern Medical University; Department of Nursing; Shenzhen; China

Basic information about other authors:

XiaoJiao Wang Southern Medical University; School of Nursing; Guangzhou; China

Chun Zhao Southern Medical University; School of Nursing; Guangzhou; China

ZongLian Guo Shenzhen Hospital, Southern Medical University; Department of Obstetrics; Shenzhen; China

Jue Li Shenzhen Hospital, Southern Medical University; Department of Nursing; Shenzhen; China

Fang Yang Shenzhen Maternity and Child Healthcare Hospital; Department of Obstetrics; Shenzhen; China

Corresponding author:

WenZhi Cai Shenzhen Hospital, Southern Medical University, Xinhu Road 1333, Baoan District, 518101 Shenzhen, China; Email: caiwzh@smu.edu.cn; Tel: +86-14706741685

Key Words: Jaundice, Neonate; Mothers; Health Knowledge, Attitudes, Practice; foctor

Word count (excluding title page, abstract, literature, drawings and tables): 3262

For peer review only

ABSTRACT

Objective: This study aimed to assess knowledge, attitudes and practices related to neonatal jaundice among mothers in Shenzhen, China and analyse associated factors.

Design: A cross-sectional study.

Setting: This study was conducted in Shenzhen Hospital, Southern Medical University, a university-affiliated, tertiary level-A, public hospital in China. On average, 4000 mothers are discharged from this hospital after childbirth each year, most of whom have access to a mobilephone and the Internet[1].

Participants: Participants were 403 healthy mothers discharged from the study hospital from April 2021 to June 2021. Participants were recruited using convenience sampling.

Primary outcome: Mothers' knowledge, attitudes and practices related to neonatal jaundice, modelled using binary logistic regression.

Secondary outcomes: Factors associated with mother's knowledge, attitudes and practices related to neonatal jaundice.

Results: The questionnaire was reliable (Cronbach's alpha=0.802) and valid (scale-level content validity index=0.958) and the valid response rate was 96.4%. Only 46.4% of participatingmothers had good knowledge about neonatal jaundice and only 41.7% indicated they would seek information about neonatal jaundice. A binary logistic regression analysis showed good knowledge about jaundice was associated with a high education level (i.e., master's degree or above; odds ratio [*OR*]=5.977, 95% confidence interval [*CI*]: 1.994~17.916, *P*=0.001), prioreducation on neonatal jaundice (*OR*=3.617, 95%*CI*: 1.637~7.990, *P*=0.001), and male babies (*OR*=1.714, 95%*CI*: 1.122~2.167, *P*=0.013). A positive attitude toward jaundice was associated with being cared for by a '*yuesao*' (*OR*=1.969, 95%*CI*: 1.264~3.066, *P*=0.003) and good knowledge about jaundice (*OR*=1.804, 95%*CI*: 1.194~2.726, *P*=0.005). Finally, good practices related to neonatal jaundice were associated with prior education on neonatal jaundice (*OR*=2.260, 95%*CI*: 1.105~4.625, *P*=0.026) and good knowledge about jaundice (*OR*=3.112, 95%*CI*: 2.040~4.749, *P*<0.001).

Conclusion: Many mothers have poor knowledge about jaundice, especially regarding causes,danger signs, and breast milk jaundice. Maternal information seeking behaviour about neonatal jaundice needs to be improved. Medical staff should incorporate information about the causes, danger signs of jaundice and breast milk jaundice in maternal health education. It is also necessary to strengthen health education for mothers, especially those with low education and no *yuesao*, and provide reliable websites where mothers can obtain information about neonatal jaundice.

Strengths and limitations of this study

For peer review only

- A strength of this study was that participants were mothers with healthy infants who had been discharged from birth hospital; these mothers may be more likely to ignore the problem of jaundice.
- The investigation time was the peak period of jaundice occurrence, which aimed to avoid recall bias.
- This study was conducted in a single health facility, meaning the findings are not broadly generalisable.
- This study only focused on new mothers and did not include significant others who may have roles in care of the newborn (e.g. grandmothers, sisters or aunts).

INTRODUCTION

Neonatal jaundice, also known as neonatal hyperbilirubinemia, refers to yellow staining of the skin or other organs caused by the accumulation of bilirubin in the body[2]. It is a common clinical problem in the neonatal period, and approximately 50%~60% of full-term infants and 80% of premature infants develop jaundice within 1 week after birth[3]. In many infants, neonatal jaundice is a benign condition. However, severe hyperbilirubinemia may cause acutebilirubin encephalopathy(ABE) or kernicterus, which may progress to nerve deafness, choreoathetoid cerebral palsy, intellectual disability and even death[4-5]. A report from China showed that from January to December 2009, 348 cases of kernicterus were recorded in 33 hospitals[6]. In addition, a worldwide survey reported that at least 480,700 newborns develop severe hyperbilirubinemia each year, with the risk for kernicterus being 13% (n=75,400) and that for death being 24% (n=114,100)[7]. Therefore, neonatal jaundice is a serious threat to the life and health of newborns, and the associated high rates of disability and mortality place heavy burdens on society and families.

Early detection and timely treatment of neonatal jaundice are key strategies to prevent ABE and kernicterus. However, neonatal jaundice generally peaks on the 5th–7th day after birth[2],at which time most healthy full-term infants have been discharged from hospital. Therefore, most neonatal jaundice occurs at home. As the main caregivers of newborns after discharge from hospital, mothers are often the first caregivers to observe jaundice, its progression, and early signs of ABE and kernicterus and are central to achieving favourable outcomes for management of neonatal jaundice. Wennberg[8] et al reported that providing mothers with detailed information about neonatal jaundice and its risks was associated with a reduction in the incidence of ABE in Nigeria. The Stop Kernicterus in Nigeria(SKIN) consortium[9]demonstrated that a delay in careseeking, regardless of birth site, is a major contributor to ABE and kernicterus, proposed that maternal education on neonatal jaundice should be targeted as an intervention strategy. The clinical practice guidelines for neonatal jaundice from National Institute for Health and Care Excellence[10] and American Academy of Pediatrics Subcommittee[3] also recommend that parents and caregivers are educated about neonatal jaundice, especially on how to check their baby for jaundice and what to do when jaundice is suspected. These suggest that maternal jaundice instruction be given high priority

For peer review only

among the myriad topics. However, effective instruction starts with meaningful engagement between hospital staff and mothers[11]. This highlights the hospital staff need to clarify what mothers know about jaundice and their current attitudes and practices, which will allow health education programmes to target identified gaps. In China, no evaluation tools and reports related to maternal knowledge, attitudes and practices about neonatal jaundice are available.

Therefore, this study designed a questionnaire to assess knowledge, attitudes and practices related to neonatal jaundice among mothers in Shenzhen, China. The information obtained maybe used to assist healthcare providers in designing educational programmes to improve awareness about neonatal jaundice among mothers, which will ultimately help to prevent disabilityand deaths among newborns.

METHODS

Study setting and design

We conducted an anonymous, self-administered, cross-sectional survey involving 403 mothers in Shenzhen, China from April to June 2021. Participants were recruited using convenience sampling.

Study population

The target population was mothers who gave birth at Shenzhen Hospital, Southern Medical University, China from April to June 2021.

Inclusion and exclusion criteria

Mothers were eligible if they were discharged from the hospital without serious illness after childbirth, and had access to a mobile phone and the Internet. Mothers were excluded if they were not the main caregiver of their newborn after being discharged from the hospital or could not complete the questionnaire by themselves. Moreover, we excluded mothers whose newborns were admitted to the neonatal intensive care unit for treatment or that died after birth.

Ethical considerations

Ethical clearance was obtained from the Ethical Review Committee of Shenzhen Hospital of Southern Medical University (approval number: NYSZYYEC20210004). Data were collected from each participant after they received a clear explanation of the purpose and importance of this study and provided informed consent. Participating mothers were informed that participation was fully voluntary and they could withdraw from the study at any time or refuse to answer any question. They were also informed they could ask for clarification about anyaspect of the study and that the study would not cause harm. Participants did not receive any monetary incentive to participate in this study. All personal information was de-identified and kept securely, and every effort was made to maintain participants' confidentiality. Furthermore, after the investigation, each mother was informed about

For peer review only

seeking healthcare from a nearby clinic immediately if any signs of jaundice were identified via mobile phone text messages.

Data collection tools

Data were collected using a questionnaire that covered baseline characteristics, knowledge, attitudes and practices regarding neonatal jaundice. These items were developed with reference to: 1) an established guideline on neonatal jaundice[10], 2) an integrative review[12], and 3) three services for investigating maternal knowledge, attitude and behaviour regarding neonatal jaundice[13-15]. We also consulted seven neonatologists and discussed the items among the research team. The draft questionnaire was pretested with 20 mothers to ensure the items were appropriate and clear. The final version of the questionnaire included 27 items. Sixteen items evaluated knowledge (categorical responses: 'true', 'false' and 'do not know'), seven items assessed attitude (categorical responses: 'strongly agree', 'agree', 'not sure', 'disagree', 'strongly disagree'), and four items covered practices (yes/no responses).

Validity and reliability

Content validity of the questionnaire was appraised quantitatively by sending the final version of the questionnaire to a group of experts including 7 specialists in the field of neonatology. On the basis of the experts' feedbacks about relevancy, the instrument S-CVI was calculated as 0.958, which was higher than the value of 0.8 that indicates adequate validity[16].

Cronbach's alpha[17] was used to check the reliability of the questionnaire, which is the most common measure of internal consistency. In this study, the Cronbach's alphas were 0.802 for the whole questionnaire, 0.789 for the knowledge dimension, 0.721 for the attitude dimensionand 0.414 for the practice dimension. The Cronbach's alphas for the knowledge and attitude dimensions were higher than 0.7, which were within the acceptable recommended range. However, that for practice (0.414) was below 0.7[18]. The small number of items (four items) may explain the low alpha estimate for this dimension. To avoid the impact of a small number of items, Cronbach[19] proposed the mean inter-item correlation (ρ) in 1951 to estimate the internal consistencyfor the dimensions with a small number of items. Generally, a mean inter-item correlation (ρ) between 0.15 and 0.20 indicates acceptable internal consistency [20]. In this study,the mean inter-item correlation was 0.15 for the practice dimension, which was within the acceptable range.

Data collection procedures

On the day each mother was discharged after delivery, the investigator informed them of the purpose, duration and method of the study, and obtained their phone number. About 5 days after discharge from the hospital, the investigator sent the questionnaire link, which was developed using the 'Question star' platform, to participating mothers via mobile phone and then called the participant to complete the questionnaire on the same day. In total, 413 questionnaires were completed. We excluded 10 participants: three were not completed, three wrote their names instead of their age, two had missing data for age, one wrote her age as '240', and one participants gave

For peer review only

obviously unreasonable responses. This left 403 valid questionnaires for analysis, giving an effective recovery rate of 96.4%. Detailed data collection procedures are presented in Figure 1.

Data processing and analysis

We used SPSS version 25.0 (IBM Corp., Armonk, NY) for all data analyses. Descriptive statistics were calculated for baseline characteristics and categorical variables using simple frequencies and percentages. The main outcome variables were knowledge, attitudes and practices. The knowledge score for each participant was determined by allotting a score of '1' to correct responses and '0' (zero) to incorrect and 'do not know' responses. Therefore, the maximum obtainable knowledge score was 16. A knowledge score ≤10 was considered poor knowledge, and scores >10 represented good knowledge[21]. The attitude scores were obtained by assigning points to responses on the 5-point Likert scale (1 point for 'strongly disagree' to 5 points for 'strongly agree'). The maximum obtainable attitude score was 35 points. An attitude score of ≤28 was considered a poor attitude and scores >29 were categorised asa good attitude[21]. Similarly, the practice score for each participant was determined by allotting a score of '1' to correct responses and '0' (zero) to incorrect responses. This gave a maximum obtainable practice score of 4. Those with a practice score of <4 were regarded as having poor practice, while a score of 4 was considered to reflect good practice[21]. Finally, the levels of knowledge, attitude and practice were coded as 0 for poor, 1 for good[21].

Chi-square tests (bivariable analyses) were used to determine the association between the baseline and outcome variables. All variables with P<0.05 in the bivariable analysis were included in the binary logistic regression (multivariable analysis) to determine the associations between dependent (knowledge, attitudes and practices) and independent(baseline characteristics) variables. Statistical significance was represented by *P* <0.05 at a 95% confidence level.

Patient and public involvement

Before the formal survey, the researchers interviewed 20 mothers to determine the readability of the questionnaire, the time required and the best way to conduct the investigation.

RESULTS

Participants' baseline characteristics

Of the 403 mothers included in our analyses, a majority were aged 28–32 years (48.9%) and 75.7% had a spontaneous vaginal delivery. Most mothers (80.6%) had received prior health education about jaundice, and most had not experienced their infants being treated for jaundice (previous infants: 86.1%; present infant: 72.0%). Participants' baseline characteristics are presented in Table 1.

Mothers' knowledge of neonatal jaundice

For peer review only

We found that 45.4% of participating mothers had good knowledge regarding neonatal jaundice. The rates of correct answers of the knowledge questions ranged from 29% to 96.8%. The questions that showed low rates of correct answers were: 'Cranial haematoma may cause neonatal jaundice' (29%), 'Blood test is the gold standard for diagnosing neonatal jaundice' (34.2%), 'It is abnormal for jaundice to appear within 24 hours after birth' (36.2%), 'It is abnormal for jaundice to reappear after it subsides' (37%), and 'Breast milk jaundice is a benign and self-limited condition, where interruption of breast feeding is not recommended as a therapeutic intervention' (37%). Table 2 presents scores for knowledge about neonatal jaundice among participating mothers.

Mothers' attitudes towards neonatal jaundice

The results revealed that 42.2% of participating mothers had poor attitudes towards neonatal jaundice. Over half of the participants strongly agreed that post-discharge observation was necessary and post-partum visits were needed to measure the bilirubin level (52.4% and 51.9%, respectively). However, some mothers believed that neonatal jaundice was a common physiological phenomenon that would not cause serious consequences (10.9%), and 29.2% lacked understanding that adequate feeding was conducive to resolving jaundice. Mothers' attitudes towards neonatal jaundice are presented in Table 3.

Mothers' practices regarding neonatal jaundice

In general, 53.1% of mothers had good practices regarding neonatal jaundice, and 96% checked their baby for jaundice after discharge. However, only 41.9% indicated they would take the initiative to learn about neonatal jaundice after discharge. Mothers' practices regarding jaundice are shown in Table 4.

Factors associated with knowledge, attitudes and practices related to neonatal jaundice among mothers

The binary logistic regression analysis revealed that good knowledge about jaundice was associated with a high level of education (master's degree or above; odds ratio [*OR*]=5.977, 95% confidence interval [*CI*]: 1.994~17.916, P=0.001), receiving prior health education on neonatal jaundice (*OR*=3.617, 95%*CI*: 1.637~7.990, *P*=0.001), and male babies (*OR*=1.714, 95%*CI*: 1.122~2.167, *P*=0.013). A positive attitude towards jaundice was associated with being cared for by a '*yuesao*' (matron specialised in maternal and newborn care) (*OR*=1.969, 95%*CI*: 1.264~3.066, *P*=0.003) and good knowledge about jaundice (*OR*=1.804, 95%*CI*: 1.194~2.726, *P*=0.005). Finally, good practices related to jaundice were associated with prior health education on neonatal jaundice (*OR*=2.260, 95%*CI*: 1.105~4.625, *P*=0.026) and good knowledge about jaundice (*OR*=3.112, 95%*CI*: 2.040~4.749, *P*<0.001). Factors associated with knowledge, attitudes and practices related to neonatal jaundice among mothers are shown in Table 5

For peer review only

DISCUSSION

The majority (80.6%) of participants in this study had received prior health education on neonatal jaundice, which suggested that their knowledge about neonatal jaundice would be high. However, this study found that many mothers had poor knowledge regarding neonatal jaundice, and only 45.5% of participating mothers had good knowledge about neonatal jaundice. This may be because health workers were not adequately informed about neonatal jaundice, which suggested it is necessary to intensify efforts to improve knowledge about neonatal jaundice among health workers in China to provide better maternal instruction on neonatal jaundice, perhaps through in-service training on a scheduled basis.

The present study reported that many mothers had poor knowledge regarding the causes and danger signs of jaundice, and breast milk jaundice. Relatively few mothers provided correct answers to some items; for example, ‘Cranial haematoma may cause neonatal jaundice’ (29%), ‘Blood test is the gold standard for diagnosing neonatal jaundice’ (34.2%), ‘It is abnormal for jaundice to appear within 24 hours after birth’ (36.2%), ‘It is abnormal for jaundice to reappear after it subsides’ (37%), and ‘Breast milk jaundice is a benign and self-limited condition, where interruption of breast feeding is not recommended as a therapeutic intervention’ (37%). Poor knowledge about the causes and danger signs of jaundice may mean mothers turn to traditional treatments, which results in delays in seeking medical attention for neonatal jaundice, thereby contributing to the development of ABE and kernicterus[22]. Having poor knowledge of breast milk jaundice may also mean mothers discontinue breastfeeding after jaundice occurs; however brief, such discontinuation may jeopardise an infant’s ability to return to exclusive breastfeeding, which is unnecessarily harmful to the infant and traumatic for mothers[23]. Therefore, it is recommended that medical staff incorporate information about the causes and danger signs of jaundice, and breast milk jaundice into neonatal jaundice health education programmes.

Importantly, our study highlighted that maternal information seeking behaviour related to neonatal jaundice needs to be improved. Only 41.7% of mothers in this study indicated they took the initiative to seek information about neonatal jaundice. This poor practice could be because physical and psychological changes in the postpartum period mean mothers’ energy is limited. However, it could also be attributable to low health literacy. A previous study[24] reported that the lower the parents’ health literacy, the less likely they were to take the initiative to obtain information about their child’s health. Actively

For peer review only

understanding relevant knowledge will help to improve maternal awareness of neonatal jaundice, which will be conducive to managing neonatal jaundice after discharge from hospital. In addition, with the popularisation of the Internet and smart phones, more and more parents are using these ways to access parenting knowledge; however, they complain that they face major challenges in identifying whether the information is reliable[24, 25]. Therefore, it is recommended that when providing education about neonatal jaundice, medical staff also provide mothers with some reliable websites to facilitate the active information seeking about neonatal jaundice.

Our multivariate analysis revealed that mothers who had a male infant were more likely be knowledgeable about neonatal jaundice compared with mothers who had a female infant. This may be because male infants are more likely to manifest jaundice[26]. Having received prior education on neonatal jaundice from medical staff and a high education level were both determinants of knowledge about neonatal jaundice. This finding was consistent with the results of a study from Egypt[14] that confirmed it is necessary and effective for medical staff to provide information about jaundice to mothers, especially those with lower education levels.

Our multivariate analysis of attitudes revealed that mothers who were cared for by ‘yuesao’ were more likely to have a positive attitude toward neonatal jaundice than other mothers. Chinese tradition dictates that new mothers stay home and rest for a ‘confinement period’ of about 1 month (28–42 days) after giving birth, which is thought to facilitate recovery. Previous studies reported that being cared for by a *yuesao* during this stage can help improve the health of mothers and babies[27, 28], reduce postpartum depression[29, 30], and facilitate breastfeeding[31]. Therefore, hiring a *yuesao* to prepare post-partum foods and help with household and childcare tasks has become increasingly popular in many urban families. Our study showed that the mothers who were cared for by a *yuesao* were more likely to respond positively to neonatal jaundice. This may be because the *yuesao* offers a valuable resource for jaundice counselling. Therefore, it is important that institutions that train *yuesao* should cover dealing with common problems such as neonatal jaundice as well as maternal and child care skills.

Our multivariate analysis of practices related to neonatal jaundice revealed that mothers who had received prior education on neonatal jaundice from medical staff were more likely to have good practices related to

jaundice than other mothers. This finding was consistent with a study conducted in Nigeria[32] that showed mothers who obtained knowledge about neonatal jaundice from medical staff were significantly less likely to self-treat, and were more likely to seek medical treatment promptly. We also found that mothers with good knowledge about neonatal jaundice were more likely to have good attitudes and practices; this was consistent with the 'KAP' model[33], which suggests greater knowledge is the basis for good attitudes and practices.

CONCLUSION

Overall, mothers' knowledge about jaundice was low, especially regarding cause, danger signs, and breast milk jaundice. Active information seeking behaviour about neonatal jaundice needs to be improved. Therefore, it is recommended that medical staff incorporate information about the causes, danger signs, and breast milk jaundice into neonatal jaundice health education programmes, and provide reliable websites for mothers to obtain information about neonatal jaundice. This study also showed that the mother's education level was an important factor that is significantly associated with knowledge about jaundice. In addition, mothers receiving care from a *yuesao* tend to have positive attitudes toward jaundice. Enhancing jaundice-related education programmes targeting mothers with a low education level and no *yuesao* care is important.

Acknowledgments: We would like to acknowledge seven neonatologists and my research team for giving feedback to the questionnaire development. Our appreciation also goes to the study participants for generously spending time and providing information in this survey.

Contributors: Y.H. and L.C. are joint first authors. Y.H., L.C., and X.J.W. designed the study. Y.H., X.J.W., C.Z., Z.L.G., F.Y., and J.L. collected the data. Y.H., L.C., and X.J.W. analyzed the data. HY drafted the manuscript. W.Z.C., Y.H. and L.C. contributed to the interpretation of the results and critical revision of the manuscript for important intellectual content and approved the final version of the manuscript. All authors have read and approved the final manuscript. W.Z.C.,Y.H.,and L.C.are the study guarantors.

Funding: This study was supported by grants from Sanming Project of Medicine in ShenZhen, China (SZSM201612018).

Competing interests: None declared.

For peer review only

Patient consent for publication: Not required.

Data availability statement: All data relevant to the study are included in the article or uploaded as supplementary information.

REFERENCES

1. Number of mobile cell phone subscriptions in China from September November 2019 to November 2020. *Statista* 2021.

2. Wang WP. Pediatrics. Poeple's Medical Publishing House. 2018.

3. Management of hyperbilirubinemia in the newborn infant 35 or more weeks of gestation. *Pediatrics* 2004;114(1):297-316.

4. Riordan SM, Shapiro SM. Review of bilirubin neurotoxicity I: molecular biology and neuropathology of disease. *Pediatr Res* 2020;87(2):327-31.

5. Karimzadeh P, Fallahi M, Kazemian M, et al. Bilirubin Induced Encephalopathy. *Iran J Child Neurol*. 2020;14(1):7-19.

6. Ma XL, Shi LP, Du LZ, et al. Clinical characteristics of bilirubin encephalopathy in Chinese newborn infants-a national multicenter survey. *Zhonghua Er Ke Za Zhi* 2012;50(05): 331-335.

7. Bhutani VK, Zipursky A, Blencowe H, et al. Neonatal hyperbilirubinemia and Rhesus disease of the newborn:incidence and impairment estimates for 2010 at regional and global levels. *PEDIATRRES* 2013;74:86-100.

8. Wennberg RP, Oguche S, Imam Z, et al. Maternal Instruction About Jaundice and the Incidence of Acute Bilirubin Encephalopathy in Nigeria. *J Pediatr* 2020;221:47-54.

9. Dlala UM, Wennberg RP, Abdulkair I, et al. Patterns of acute bilirubin encephalopathy in Nigeria: a multicenter pre-intervention study. *J Perinatol* 2018;38(7):873-880.

10. UK NIFH. Addendum to Jaundice in newborn babies under 28 days. London: National Institute for Health and Care Excellence (UK). 2016.

11. Watchko JF. Maternal Instruction on Neonatal Jaundice: What Can we Learn from the Stop Kernicterus in Nigeria (SKIN) Experience? *J Pediatr* 2020;221:7-8.

12. Editorial Board of Chinese Journal of Pediatrics Subspecialty Group of Neonatology, The Society of Pediatrics, Chinese Medical Association. [Experts consensus on principles for diagnosis and treatment of

For peer review only

neonatal jaundice]. *Zhonghua Er Ke Za Zhi* 2010;48:685–6.

13. Adoba P, Ephraim RKD, Kontor KA, et al. Knowledge Level and Determinants of Neonatal Jaundice: A Cross-Sectional Study in the Effutu Municipality of Ghana. *Int J Pediatr* 2018;3901505.

14. Moawad EMI, Abdallah EAA, Ali YZA. Perceptions, practices, and traditional beliefs related to neonatal jaundice among Egyptian mothers. *Medicine* 2016;95(36):e4804.

15. Demis A, Getie A, Wondmieneh A ,et al. Knowledge on neonatal jaundice and its associated factors among mothers in northern Ethiopia: a facility-based cross-sectional study. *BMJ Open* 2021;11(3):e44390.

16. Shi JC, Mo XK, Sun ZQ. Content validity index in scale development. *Zhong Nan Da Xue Xue Bao Yi Xue Ban* 2012;37(02): 49-52.

17. McNeish D. Thanks coefficient alpha, we'll take it from here. *Psychol Methods* 2018;23(3):412-33.

18. Li Z. Nursing Research Methods[M]. Poeple's Medical Publishing House. 2018.

19. Cronbach LJ. Coefficient alpha and the internal structure of tests. *Psychometrika* 1951;16(3):297-334.

20. Clark LAWD. Constructing validity: Basic issues in objective scale development. *Psychol Assess* 1995;7(3):309-19.

21. Li TT, Wang J, Chen X, Chen L, et al.Obstetric Nurses' Knowledge, Attitudes, and Professional Support Related to Actual Care Practices About Urinary Incontinence. *Female Pelvic Med Reconstr Surg* 2021;27(2):e377-84.

22. Salia SM, Afaya A, Wuni A, et al. Knowledge, attitudes and practices regarding neonatal jaundice among caregivers in a tertiary health facility in Ghana. *PLoS One* 2021;16(6):e0251846.

23. Preer GL, Philipp BL. Understanding and managing breast milk jaundice. *Arch Dis Child Fetal Neonatal Ed* 2011;96(6):F461-6.

24. Kubb C, Foran HM. Online Health Information Seeking by Parents for Their Children: Systematic Review and Agenda for Further Research. *J Med Internet Res* 2020;22(8):e19985.

25. Smith CA, Parton C, King M, et al. Parents' experiences of information-seeking and decision-making regarding complementary medicine for children with autism spectrum disorder: a qualitative study. *BMC Complement Med Ther* 2020;20(1):4.

26. Guidelines QC. Neonatal jaundice, 2019.

27. Li YY, Wang HY. The impact of yuesao on the health of mothers and infants. *Chinese Jounal Of*

For peer review only

PracticalActical Nursing 2010;26(27): 42-43.

28. Yang L,Zhu MY, Yin Y. Impact of babysitter's care on health status of infants: a survey study. *Journalof Nursing Science* 2011;(18): 87-88.

29. Xing CG. Practical Strategies and Policy Implications of Postpartum Doulas' Intervention on Postpartum Depression. *Medicine and Society* 2021;34(04):11-16.

30. Peng K, Zhou L, Liu X, et al. Who is the main caregiver of the mother during the doing-the-month: is there an association with postpartum depression? *BMC Psychiatry* 2021;21(1):270.

31. Wu W, Zhang J, Silva ZI,et al. Factors influencing breastfeeding practices in China: A meta-aggregationof qualitative studies. *Matern Child Nutr* 2021:e13251.

32. Ezeaka CV, Ugwu RO, Mukhtar-Yola M, et al. Pattern and predictors of maternal care-seeking practices for severe neonatal jaundice in Nigeria: a multi-centre survey. *BMC Health Serv Res* 2014;14:192.

33. Badran IG. Knowledge, attitude and practice the three pillars of excellence and wisdom: a place in themedical profession. 1995.

Figure Legend

Figure 1 The detailed data collection procedures for the survey.

Table 1. Baseline characteristics of participating mothers (N=403)

Variables	Characteristics	N（%）
**Sociodemographic data**		
Age(years)	19~27	114（28.3）
	28~32	197（48.9)
	33~45	92（22.8）
Blood group	O	145（36.0）
	A	121（30.0）
	B	106（26.3）
	AB	31（7.7）
Education level	High school and below	68（16.9）

For peer review only

	University	310（76.9）
	Postgraduate and above	25（6.2）
Occupation	Employed	267（66.3）
	Self-employed	40（9.9）
	Housewife	82（20.3）
	Others	14（3.5）
Average family monthly income (RMB)	≤5000	50（12.4）
	5001~10000	154（38.2）
	10001~20000	125（31）
	20001~30000	35（8.7）
	≥30001	39（9.7）
Time from the place of residence to the delivery hospital (minutes)	≤10	52（12.9）
	10~30	212（52.6）
	30~60	126（31.3）
	≥60	13（3.2）
**Delivery history**		
Parity	Primiparous	224（55.6）
	Multipara	179（44.4）
Delivery mode	Spontaneous vaginal	305（75.7）
	Caesarean section	98（24.3）
**Infant's Information**		
Sex	Male	210（52.1）
	Female	193（47.9）
Birth weight[1]	Low	365（7.7）
	Normal	31（90.6）
	Hight	7（1.7）
Feeding way	Exclusive breastfeeding	196（48.6）
	Mixed feeding	197（48.9）
	Exclusive Formula-feeding	10（2.5）
Cranial hematoma[2]	Yes	15（3.7）
	No	364（90.3）
	Not sure	24（6.0）

For peer review only

Whether to pass meconium within 24 hours	Yes	397（98.5）
	No	6（1.5）
Predischarge bilirubin level	Normal	312（77.4）
	Hight	91（22.6）
"yuesao" [3]	Yes	138（34.2）
	No	265（65.8）
**Prior experience regarding neonatal jaundice**		
Prior health education on neonatal jaundice	Yes	361（80.6）
	No	42（10.4）
Previous child history of neonatal jaundice	Yes	56（13.9）
	No	347（86.1）
Current child admitted to thehospi tal for treatment due to jaundice after discharge	Yes	113（28.0）
	No	290（72.0）
Mother's knowledge level	good	183（45.4）
	poor	220（54.6）
Mother's attitude level	good	170（42.2）
	poor	233（57.8）
Mother's practice level	good	214（53.1）
	poor	183（46.9）

Note: [1.]Weight: low weight <2500 g; normal weight 2500–4000 g; high weight >4000 g. [2.]Cranial haematoma: haematoma caused by rupture and bleeding of subperiosteal v essels in the parieto-occipital region due to birth injury. [3.]*yuesao*: a maternity matron specialised in caring for mothers and newborn infants.

For peer review only

Table 2. Maternal knowledge about neonatal jaundice (N=403)

**Items**	**Ture N（%）**	**False N（%）**	**Don't know N（%）**	**Correct rate N（%）**
**Observation of neonatal jaundice**				
When newborns develop jaundice, the ir skin will turn yellow.	390（96.8）	4（1.0）	9（2.2）	390（96.8）
When newborns develop jaundice, their	344（85.4）	12（3.0）	47（11.7）	344（85.4）

face will turn yellow firstly.				
When looking for jaundice, check the naked baby in bright and preferably n atural light.	365（90.6）	10（2.5）	28（6.9）	365（90.6）
**Classification of neonatal jaundice**				
Neonatal jaundice is divided into physi ological jaundice and pathological jaun dice.	371（92.1)	3（0.7）	29（7.2）	371（92.1)
**Danger signs of neonatal jaundice**				
Palms and soles turn yellow, indicatin g that jaundice is severe.	265（65.8）	29（7.2）	109（27.0）	265（65.8)
It is an abnormal condition if the jaun dice appears within first 24 h.	146（36.2）	155（38.5）	102（25.3）	146（36.2)
It is an abnormal condition that the ja undice reappears after it has subsided.	149（37.0）	143（35.5）	111（27.5）	149（37.0）
**Complication of neonatal jaundice**				
Severe jaundice may lead to brain da mage.	325 80.6）	7（1.7）	71（17.6）	325（80.6）
**Cause of neonatal jaundice**				
The mother's blood type is O, and the father's blood type is A, B, or AB, w hich may cause neonatal jaundice.	228（56.6）	38（9.4）	137（34.0）	228（56.6）
Cranial hematoma may cause neonatal jaundice.	117（29.0）	39（9.7）	247（61.3）	117（29.0）
Bowel obstruction may cause neonatal jaundice.	281（69.7）	14（3.5）	108（26.8）	281（69.7）
Broad bean disease(G6PD) may cause jaundic.	163（40.4）	25（6.2）	215（53.3）	163（40.4）
Breastfeeding may cause jaundice	200（49.6）	109（27.0）	94（23.3）	200（49.6）
**Breast milk jaundice**				
Breast milk jaundice is a benign and s elf-limited condition, where interruption of breast feeding is not recommended as a therapeutic intervention.	149（37.0）	135（33.5）	119（29.5）	149（37.0）
**Diagnosis of neonatal jaundice**				

For peer review only

Blood test is the gold standard for diagnosing neonatal jaundice.	138（34.2）	127（31.5）	138（34.2）	138（34.2）
**Treatment of neonatal jaundice**				
Phototherapy is a common, effective and safe treatment method for neonatal jaundice.	367（91.1）	3（0.7）	33（8.2）	367（91.1）

Table 3. Maternal attitudes towards neonatal jaundice (N=403)

**Items**	**Strongly disagree N（%）**	**Disagree N（%）**	**Not sure N（%）**	**Agree N（%）**	**Strongly agree N（%）**
I think neonatal jaundice is a common physiological phenomenon and will not cause serious consequences.（-）	80（19.1）	216（53.6）	63（15.6）	39（9.7）	5（1.2）
I think that a baby with jaundice, don't need treat which will self-recovered.（-）	151（38.5）	201（49.9）	43（10.7）	6（1.5）	2（0.5）
I think it is very important to observe neonatal jaundice after discharged from the hospital.	5（1.2）	0（0.0）	6（1.5）	181（44.9）	211（52.4）
I think it is necessary for post-partum visitors to assess jaundice condition.	8（1.0）	0（0.0）	8（2.0）	182（45.2）	209（51.9）
I think a baby with suspected jaundice should go to the medical institution or the community healthcare centres to measure the bilirubin level in time.	3（0.7）	5（1.2）	10（2.5）	230（57.1）	155（37.5）
I believe that adequate breastfeeding is good for jaundice.	5（1.2）	29（7.2）	84（20.8）	196（48.6）	89（22.1）
I think it is necessary to learn the knowledge of neonatal jaundice.	2（0.5）	0（0.0）	10（2.5）	216（53.6）	175（43.4）

Note: - represents reverse scored items.

For peer review only

Table 4. Maternal practices regarding neonatal jaundice (N=403)

Items	Yes N（%）	No N（%）
I took the initiative to seek information about neonatal jaundice.	168（41.7）	235（58.3）
After discharge, I checked my infant for jaundice such as the color of the skin, sclerae 、urine 、 bowel.	387（96.0）	16（4.0）
After discharge, I followed the doctor's instructions that take the infant to the medical institution or the community healthcarecentres to measure the bilirubin level.	376（93.3）	27（6.7）
After discharge, I breastfed adequatly.	359（89.1）	44（10.9）

For peer review only

Table 5. Binary logistic regression analysis of maternal knowledge, attitudes and practices related to neonatal jaundice (N=403)

Variables	Classification	Knowledge			Attitude			Behaviour		
		OR	*95%CI*	*P*	*OR*	*95%CI*	*P*	*OR*	*95%CI*	*P*
Education level	High School and below (Ref.)			**0.001**						
	College and undergraduate course	3.011	1.563~5.800	0.001		*NI*			*NI*	
	Postgraduate student or above	5.977	1.994~17.916	0.001						
Occupation	Employed (Ref.)			0.110						
	Self-employed	0.403	0.183~0.887	0.024		*NI*				
	Housewives	0.730	0.409~1.302	0.286					*NI*	
	Other	1.266	0.401~3.994	0.688						
Parts	Primiparous (Ref.)		*NI*						*NI*	
	Multipara				1.498	0.995~2.255	0.053			
Prior education on neonatal jaundice	No (Ref.)									
	Yes	3.617	1.637~7.993	**0.001**		*NI*		2.260	1.105~4.625	**0.026**
Neonatec gender	Female (Ref.)									
	Male	1.714	1.122~2.617	**0.013**		*NI*			*NI*	
Cranial hematoma	No (Ref.)			0.073						
	Yes	1.549	0.510~4.706	0.440		*NI*			*NI*	
	It is not clear	0.321	0.112~0.920	0.034						
"Yuesao"	No (Ref.)		*NI*						*NI*	

For peer review only

	yes		1.969	1.264~3.066	**0.003**			
Knowledge level	Poor (Ref.)	*NA*		*NI*				
	good		1.804	1.194~2.726	**0.005**	3.112	2.040~4.749	**0.000**
Attitude level	poor (Ref.)	*NI*		*NA*				
	good					1.498	0.983~2.283	0.060

Note: *NI*, not included in the final logistic regression analysis; *NA*, not applicable.

For peer review only

For peer review only

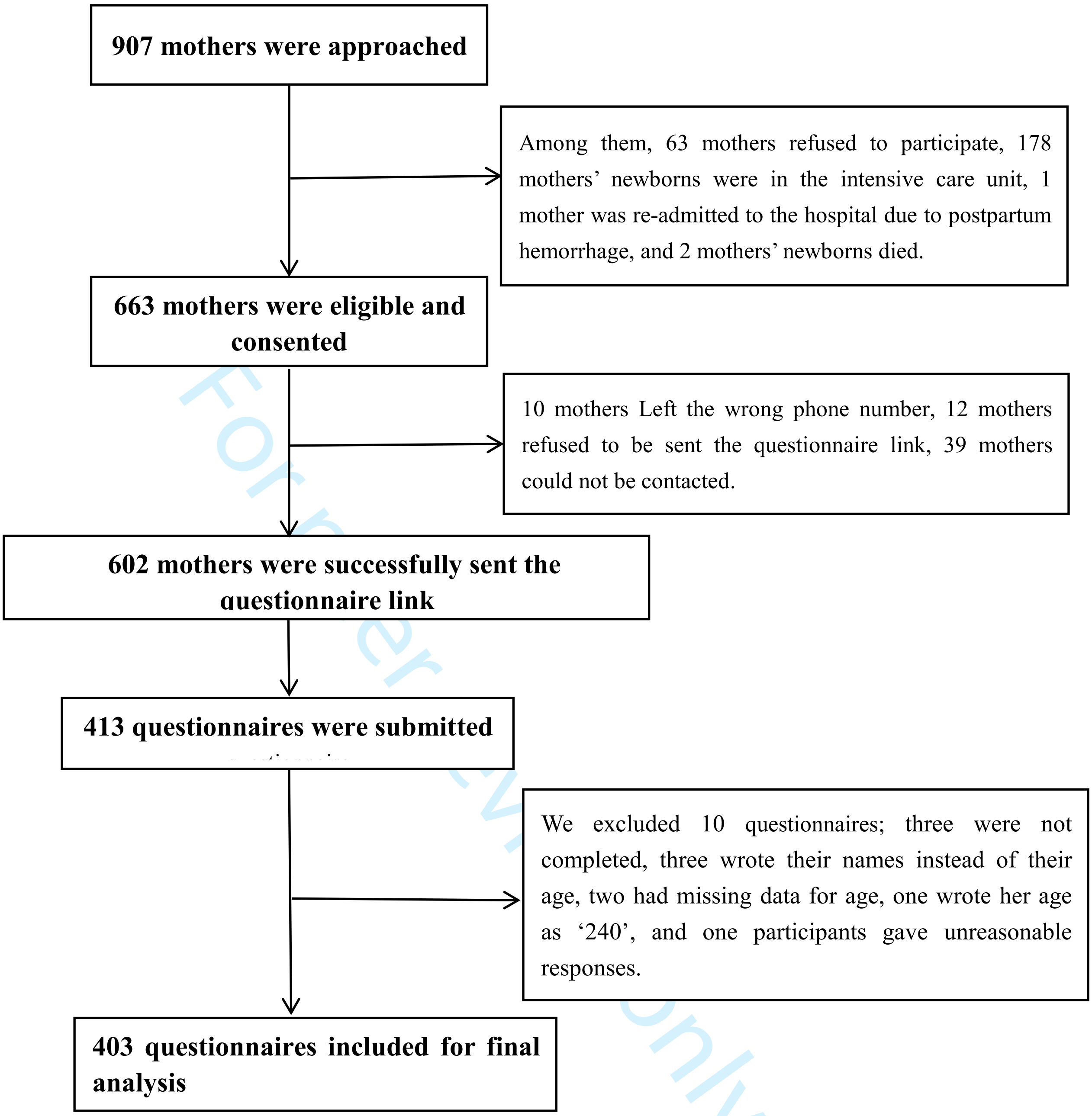

For peer review only

STROBE Statement—Checklist of items that should be included in reports of ***cross-sectional studies***

	Item No	Recommendation	Reported on page number
**Title and abstract**	1	(*a*) Indicate the study's design with a commonly used term in the title or the abstract	Page 1:title page
		(*b*) Provide in the abstract an informative and balanced summary of what was done and what was found	Page 2-3
**Introduction**			
Background/rationale	2	Explain the scientific background and rationale for the investigation being reported	Page 3-4
Objectives	3	State specific objectives, including any prespecified hypotheses	Page 4
**Methods**			
Study design	4	Present key elements of study design early in the paper	Page 4
Setting	5	Describe the setting, locations, and relevant dates, including periods of recruitment, exposure, follow-up, and data collection	Page 4 and Figure 1data collection progress
Participants	6	(*a*) Give the eligibility criteria, and the sources and methods of selection of participants	Page 4
Variables	7	Clearly define all outcomes, exposures, predictors, potential confounders, and effect modifiers. Give diagnostic criteria, if applicable	Page 6:The levels of knowledge, attitude and practice (for poor, for good)
Data sources/ measurement	8*	For each variable of interest, give sources of data and details of methods of assessment (measurement). Describe comparability of assessment methods if there is more than one group	Page 4-6
Bias	9	Describe any efforts to address potential sources of bias	Page 2-3 Strengths and limitations of this study
Study size	10	Explain how the study size was arrived at	NI:Sufficient sample size
Quantitative variables	11	Explain how quantitative variables were handled in the analyses. If applicable, describe which groupings were chosen and why	Page 6
Statistical methods	12	(*a*) Describe all statistical methods, including those used to control for confounding	Page 6
		(*b*) Describe any methods used to examine subgroups and interactions	Page 6
		(*c*) Explain how missing data were addressed	Page 5-6 excluded 10 participants' data
		(*d*) If applicable, describe analytical methods taking account of	NA

For peer review only

		sampling strategy	
		(*e*) Describe any sensitivity analyses	NA
**Results**			
Participants	13*	(a) Report numbers of individuals at each stage of study—eg numbers potentially eligible, examined for eligibility, confirmed eligible, included in the study, completing follow-up, and analysed	Figure 1data collection progres
		(b) Give reasons for non-participation at each stage	Figure 1data collection progres
		(c) Consider use of a flow diagram	Figure 1data collection progres
Descriptive data	14*	(a) Give characteristics of study participants (eg demographic, clinical, social) and information on exposures and potential confounders	Page 6 and Table1
		(b) Indicate number of participants with missing data for each variable of interest	NI
Outcome data	15*	Report numbers of outcome events or summary measures	Page 6-7
Main results	16	(*a*) Give unadjusted estimates and, if applicable, confounder-adjusted estimates and their precision (eg, 95% confidence interval). Make clear which confounders were adjusted for and why they were included	Page 7 and Table5
		(*b*) Report category boundaries when continuous variables were categorized	NI
		(*c*) If relevant, consider translating estimates of relative risk into absolute risk for a meaningful time period	NA
Other analyses	17	Report other analyses done—eg analyses of subgroups and interactions, and sensitivity analyses	NA
**Discussion**			
Key results	18	Summarise key results with reference to study objectives	Page 7-9
Limitations	19	Discuss limitations of the study, taking into account sources of potential bias or imprecision. Discuss both direction and magnitude of any potential bias	Page 2-3
Interpretation	20	Give a cautious overall interpretation of results considering objectives, limitations, multiplicity of analyses, results from similar studies, and other relevant evidence	Page 10
Generalisability	21	Discuss the generalisability (external validity) of the study results	Page 7-9
**Other information**			
Funding	22	Give the source of funding and the role of the funders for the present study and, if applicable, for the original study on which the present article is based	Page 10-11

Note: *NI*, Not included ; *NA*, Not available

For peer review only - http://bmjopen.bmj.com/site/about/guidelines.xhtml

Maternal knowledge, attitudes and practices related to neonatal jaundice and associated factors in Shenzhen, China: a facility-based cross-sectional study.

Journal:	*BMJ Open*
Manuscript ID	bmjopen-2021-057981.R1
Article Type:	Original research
Date Submitted by the Author:	18-Feb-2022
Complete List of Authors:	Huang, Ying; Shenzhen Hospital of Southern Medical University, Department of Nursing<br>Chen, Ling; Shenzhen Hospital of Southern Medical University, Department of Nursing<br>Wang, Xiaojiao; Shenzhen Hospital of Southern Medical University, Department of Nursing<br>Zhao, Chun; Shenzhen Hospital of Southern Medical University, Department of Nursing<br>Guo, Zonglian; Shenzhen Hospital of Southern Medical University, Department of Obstetrics<br>Li, Jue; Shenzhen Hospital of Southern Medical University, Department of Nursing<br>Yang, Fang; Shenzhen Maternity and Child Healthcare Hospital, Department of Obstetrics<br>Cai, Wenzhi; Shenzhen Hospital of Southern Medical University,
**Primary Subject Heading**:	Health informatics
Secondary Subject Heading:	Health services research, Health informatics, Public health
Keywords:	NEONATOLOGY, MEDICAL EDUCATION & TRAINING, Child protection < PAEDIATRICS

SCHOLARONE™ Manuscripts

I, the Submitting Author has the right to grant and does grant on behalf of all authors of the Work (as defined in the below author licence), an exclusive licence and/or a non-exclusive licence for contributions from authors who are: i) UK Crown employees; ii) where BMJ has agreed a CC-BY licence shall apply, and/or iii) in accordance with the terms applicable for US Federal Government officers or employees acting as part of their official duties; on a worldwide, perpetual, irrevocable, royalty-free basis to BMJ Publishing Group Ltd ("BMJ") its licensees and where the relevant Journal is co-owned by BMJ to the co-owners of the Journal, to publish the Work in this journal and any other BMJ products and to exploit all rights, as set out in our licence.

The Submitting Author accepts and understands that any supply made under these terms is made by BMJ to the Submitting Author unless you are acting as an employee on behalf of your employer or a postgraduate student of an affiliated institution which is paying any applicable article publishing charge ("APC") for Open Access articles. Where the Submitting Author wishes to make the Work available on an Open Access basis (and intends to pay the relevant APC), the terms of reuse of such Open Access shall be governed by a Creative Commons licence – details of these licences and which Creative Commons licence will apply to this Work are set out in our licence referred to above.

Other than as permitted in any relevant BMJ Author's Self Archiving Policies, I confirm this Work has not been accepted for publication elsewhere, is not being considered for publication elsewhere and does not duplicate material already published. I confirm all authors consent to publication of this Work and authorise the granting of this licence.

For peer review only

Title: Maternal knowledge, attitudes and practices related to neonatal jaundice and associated factors in Shenzhen, China: a facility-based cross-sectional study

Basic information about the co-authors:

Ying Huang: Southern Medical University, School of Nursing, Guangzhou, China

Ling Chen: Shenzhen Hospital, Southern Medical University, Department of Nursing, Shenzhen, China

Basic information about other authors:

XiaoJiao Wang: Southern Medical University, School of Nursing, Guangzhou, China

Chun Zhao: Southern Medical University, School of Nursing, Guangzhou, China

ZongLian Guo: Shenzhen Hospital, Southern Medical University, Department of Obstetrics, Shenzhen, China

Jue Li: Shenzhen Hospital, Southern Medical University, Department of Nursing, Shenzhen, China

Fang Yang: Shenzhen Maternity and Child Healthcare Hospital, Department of Obstetrics, Shenzhen, China

Corresponding author:

WenZhi Cai: Shenzhen Hospital, Southern Medical University, Xinhu Road 1333, Baoan District, 518101 Shenzhen, China; Email: caiwzh@smu.edu.cn; Tel: +86-14706741685

Key Words: Jaundice, Neonate; Mothers; Health Knowledge, Attitudes, Practice; Doctor

Word count (excluding title page, abstract, literature, drawings and tables): 3823

For peer review only

ABSTRACT

Objective: This study aimed to assess knowledge, attitudes and practices related to neonatal jaundice among mothers in Shenzhen, China and analyse associated factors.

Design: A cross-sectional study.

Setting: This study was conducted in Shenzhen Hospital, Southern Medical University, a university-affiliated, tertiary level-A, public hospital in China. On average, 4000 mothers are discharged from this hospital after childbirth each year, most of whom can access a mobile phone and the Internet.

Participants: Participants were 403 mothers discharged from the study hospital within 48~72 hours after vaginal delivery or 96~120 hours after caesarean delivery between April and June 2021. Participants were recruited using convenience sampling.

Primary outcome: Mothers' knowledge, attitudes and practices related to neonatal jaundice, modelled using binary logistic regression.

Secondary outcomes: Factors associated with mothers' knowledge, attitudes and practices related to neonatal jaundice.

Results: The questionnaire was reliable (Cronbach's alpha=0.802) and valid (scale-level content validity index=0.958). The valid response rate was 96.4%. Only 46.4% of participating mothers had good knowledge about neonatal jaundice and 41.7% indicated they would seek information about neonatal jaundice. A binary logistic regression analysis showed good knowledge about jaundice was associated with a high education level (i.e., master's degree or above; odds ratio [*OR*]=5.977, 95% confidence interval [*CI*]: 1.994~17.916, *P*=0.001), prior education on neonatal jaundice (*OR*=3.617, 95%*CI*: 1.637~7.990, *P*=0.001), and male babies (*OR*=1.714, 95%*CI*: 1.122~2.167, *P*=0.013). A positive attitude toward jaundice was associated with being cared for by a '*yuesao*' (maternity matron specialised in caring for mothers and newborns) (*OR*=1.969, 95%*CI*: 1.264~3.066, *P*=0.003) and good knowledge about jaundice (*OR*=1.804, 95%*CI*: 1.194~2.726, *P*=0.005). Finally, good practices related to neonatal jaundice were associated with prior education on neonatal jaundice (*OR*=2.260, 95%*CI*: 1.105~4.625, *P*=0.026) and good knowledge about jaundice (*OR*=3.112, 95%*CI*: 2.040~4.749, *P*<0.001).

Conclusion: Many mothers have poor knowledge about jaundice, especially regarding causes, danger signs and breast milk jaundice. Maternal information seeking behaviour about neonatal jaundice needs to be improved. Medical staff should incorporate information about the causes/danger signs of jaundice and breast milk jaundice in maternal health education. It is also necessary to strengthen health education for mothers, especially those with low education and no *yuesao*, and provide reliable websites where mothers can obtain information about neonatal jaundice.

For peer review only

Strengths and limitations of this study

- A strength of this study was that participants were mothers with healthy infants who had been discharged from the birth hospital; these mothers may be more likely to ignore the problem of jaundice.
- The investigation time was the peak period of jaundice occurrence, which aimed to avoid recall bias.
- Recruiting mothers and then surveying them at a later time may have prompted mothers to search for information about jaundice.
- This study focused on new mothers and did not include significant others who may have roles in care of the newborn (e.g. grandmothers, sisters or aunts).

INTRODUCTION

Neonatal jaundice, also known as neonatal hyperbilirubinemia, refers to yellow staining of the skin or other organs caused by the accumulation of bilirubin in the body.[1] It is a common clinical problem in the neonatal period, and approximately 50%~60% of full-term infants and 80% of premature infants develop jaundice within 1 week after birth.[2] In many infants, neonatal jaundice is a benign condition. However, severe hyperbilirubinemia may cause acute bilirubin encephalopathy (ABE) or kernicterus, which may progress to nerve deafness, choreoathetoid cerebral palsy, intellectual disability and even death.[3-4] A report from China showed that from January to December 2009, 348 cases of kernicterus were recorded in 33 hospitals.[5] In addition, a worldwide survey reported that at least 480,700 newborns develop severe hyperbilirubinemia each year, with the risk for kernicterus being 13% (n=75,400) and that for death being 24% (n=114,100).[6] Therefore, neonatal jaundice is a serious threat to the life and health of newborns, and the associated high rates of disability and mortality place heavy burdens on society and families.

Early detection and timely treatment of neonatal jaundice are key strategies to prevent ABE and kernicterus. However, neonatal jaundice generally peaks on the 5th–7th day after birth,[1] at which time most healthy full-term infants have been discharged from hospital. Therefore, most neonatal jaundice occurs at home. As the main caregivers of newborns after discharge from hospital, mothers are often the first to observe jaundice, its progression, and early signs of ABE and kernicterus. They are central to achieving favourable outcomes for management of neonatal jaundice. Wennberg[7] et al. reported that providing mothers with detailed information about neonatal jaundice and its risks was associated with a reduction in the incidence of ABE in Nigeria. The Stop Kernicterus in Nigeria (SKIN) consortium[8] demonstrated that a delay in seeking care, regardless of birth site, was a major contributor to ABE and kernicterus and proposed that maternal education on neonatal jaundice should be targeted as an intervention strategy. The clinical practice guidelines for neonatal jaundice from the National Institute for Health and Care Excellence[9] and American Academy of Pediatrics Subcommittee[2] also recommend

For peer review only

parents and caregivers are educated about neonatal jaundice, especially on how to check their baby for jaundice and what to do when jaundice is suspected. These guidelines suggest that maternal jaundice instruction be given high priority among the myriad topics. However, effective instruction starts with meaningful engagement between hospital staff and mothers.[10] Hospital staff therefore need to clarify what mothers know about jaundice and their current attitudes and practices, which will allow health education programmes to target identified gaps. Although similar investigations have been done in other countries or regions including Ghana,[11] Accra,[12] and Egypt,[13] no evaluation tools or reports related to maternal knowledge, attitudes and practices about neonatal jaundice are available in China.

Therefore, this study designed a questionnaire to assess knowledge, attitudes and practices related to neonatal jaundice among mothers in Shenzhen, China. The information obtained maybe used to assist healthcare providers in designing educational programmes to improve awareness about neonatal jaundice among mothers, which will ultimately help to prevent disability and deaths among newborns.

METHODS

Study setting and design

We conducted an anonymous, self-administered, cross-sectional survey involving 403 mothers in Shenzhen, China from April to June 2021. Participants were recruited using convenience sampling.

Study population

The target population was mothers who gave birth at Shenzhen Hospital, Southern Medical University, China from April to June 2021.

Inclusion and exclusion criteria

Mothers were eligible for this study if they were discharged from the hospital without serious illness after childbirth and had access to a mobile phone and the Internet. Mothers were excluded if they were not the main caregiver of their newborn after being discharged from hospital or could not complete the questionnaire by themselves. Moreover, we excluded mothers whose newborns were admitted to the neonatal intensive care unit for treatment or that died after birth.

Ethical considerations

Ethical clearance was obtained from the Ethical Review Committee of Shenzhen Hospital of Southern Medical University (approval number: NYSZYYEC20210004). Data were collected from each participant after they received a clear explanation of the purpose and importance of this study and provided informed consent. Participating mothers were informed that participation was voluntary, and they could withdraw from the study at any time or refuse to answer any question. They were also informed they could ask for clarification about any

For peer review only

aspect of the study and that the study would not cause harm. Participants did not receive any monetary incentive to participate in this study. All personal information was de-identified and kept securely, and every effort was made to maintain participants' confidentiality. Furthermore, after the investigation, each mother was informed via mobile phone text messages about seeking healthcare from a nearby clinic immediately if any signs of jaundice were identified.

Data collection tools

Data were collected using a questionnaire that covered baseline characteristics, knowledge, attitudes and practices regarding neonatal jaundice. These items were developed with reference to: 1) an established guideline on neonatal jaundice,[9] 2) an integrative review,[14] and 3) three services for investigating maternal knowledge, attitude and behaviour regarding neonatal jaundice.[13, 15, 16] We also consulted seven neonatologists and discussed the items among the research team. Following the review by the expert panel, nine mothers whose babies had experienced neonatal jaundice were conveniently recruited to provide input on the importance and clarity of the questionnaire items. Mothers were asked to suggest alternative wording for existing items and identify items that required deletion and addition as necessary. Some items were modified based on this review. For example, we modified 'Have you ever learned about neonatal jaundice' to 'Prior health education on neonatal jaundice', 'G6PD deficiency' to 'Broad bean disease', and 'Serum total bilirubin is the gold standard for diagnosing neonatal jaundice' to 'Blood test is the gold standard for diagnosing neonatal jaundice'. No items were identified for deletion or addition. The draft questionnaire was then pretested with 20 mothers who were conveniently selected from the same hospital based on the study inclusion criteria and exclusion criteria. The final version of the questionnaire included 27 items. Sixteen items evaluated knowledge (categorical responses: 'true', 'false', 'do not know'), seven items assessed attitude (categorical responses: 'strongly agree', 'agree', 'not sure', 'disagree', 'strongly disagree'), and four items covered practices (yes/no responses).

Validity and reliability

The content validity of the questionnaire was appraised quantitatively by sending the final version of the questionnaire to a group of experts including seven specialists in the field of neonatology. Based on the experts' feedback about relevance, the instrument's scale-level content validity index was calculated as 0.958, which was higher than the value of 0.8 that indicates adequate validity.[17]

Cronbach's alpha[18] was used to check the reliability of the questionnaire, which is the most common measure of internal consistency. In this study, the Cronbach's alphas were 0.802 for the whole questionnaire, 0.789 for the knowledge dimension, 0.721 for the attitude dimension and 0.414 for the practice dimension. The Cronbach's alphas for the knowledge and attitude dimensions were higher than 0.7, and were within the acceptable

For peer review only

recommended range. However, that for practice (0.414) was below 0.7.[19] The small number of items (four items) on that dimension may explain the low alpha estimate. To avoid the impact of a small number of items, Cronbach[20] proposed the mean inter-item correlation (ρ) in 1951 to estimate the internal consistency of dimensions with a small number of items. Generally, a mean inter-item correlation (ρ) between 0.15 and 0.20 indicates acceptable internal consistency. [21] In this study, the mean inter-item correlation was 0.15 for the practice dimension, which was within the acceptable range.

Data collection procedure

On the day each mother was discharged after delivery, the investigator informed them of the purpose, duration and method of this study, and obtained their phone number. About 5 days after discharge from hospital, the investigator sent the questionnaire link, which was developed using the 'Question star' platform, to participating mothers via mobile phone and then called the participant to complete the questionnaire on the same day. In total, 413 questionnaires were completed. We excluded 10 participants: three did not complete the questionnaire, three wrote their names instead of their age, two had missing data for age, one wrote her age as '240' and one participant chose the first option for each question. This left 403 valid questionnaires for analysis, giving an effective recovery rate of 96.4%. Detailed data collection procedures are presented in Figure 1.

Data processing and analysis

We used SPSS version 25.0 (IBM Corp., Armonk, NY) for all data analyses. Descriptive statistics were calculated for baseline characteristics and categorical variables using simple frequencies and percentages. The main outcome variables were knowledge, attitudes and practices. The knowledge score for each participant was determined by allotting a score of '1' to correct responses and '0' (zero) to incorrect and 'do not know' responses. Therefore, the maximum obtainable knowledge score was 16. A knowledge score ≤10 was considered poor knowledge, and scores >10 represented good knowledge.[22] The attitude scores were obtained by assigning points to responses on the 5-point Likert scale (1 point for 'strongly disagree' to 5 points for 'strongly agree'). The maximum obtainable attitude score was 35 points. An attitude score ≤28 was considered a poor attitude and scores >29 were categorised as good attitudes.[22] Similarly, the practice score for each participant was determined by allotting a score of '1' to correct responses and '0' (zero) to incorrect responses. This gave a maximum obtainable practice score of 4. Those with a practice score <4 were regarded as having poor practice, whereas a score of 4 was considered to reflect good practice.[22] Finally, the levels of knowledge, attitude and practice were coded as 0 for poor, 1 for good.[22]

Chi-square tests (bivariable analyses) were used to determine the associations between the baseline and outcome variables. All variables with P<0.05 in the bivariable analysis were included in the binary logistic regression (multivariable analysis) to determine the associations between dependent (knowledge, attitudes and practices) and

For peer review only

independent (baseline characteristics) variables. Statistical significance was represented by $P<0.05$ at a 95% confidence level.

Patient and public involvement

Before the formal survey, the researchers interviewed 20 mothers to determine the readability of the questionnaire, the time required and the best way to conduct the investigation.

RESULTS

Participants' baseline characteristics

Of the 403 mothers included in our analyses, a majority were aged 28–32 years (48.9%) and 75.7% had a spontaneous vaginal delivery. Most mothers (80.6%) had received prior health education about jaundice from health workers on the day of normal discharge from the delivery hospital (48~72 hours after vaginal delivery or 96~120 hours after caesarean delivery). Among the 403 participating mothers, 113 (28%) reported their current child was admitted to the hospital for treatment due to jaundice after discharge, and 56 (13.9%) had a previous child with a history of neonatal jaundice. Participants' baseline characteristics are presented in Table 1.

Mothers' knowledge of neonatal jaundice

We found that 45.4% of participating mothers had good knowledge regarding neonatal jaundice. The rates of correct answers to the knowledge questions ranged from 29% to 96.8%. Questions that showed low rates of correct answers were: 'Cranial haematoma may cause neonatal jaundice' (29%), 'Blood test is the gold standard for diagnosing neonatal jaundice' (34.2%), 'It is abnormal for jaundice to appear within 24 hours after birth' (36.2%), 'It is abnormal for jaundice to reappear after it subsides' (37%), and 'Breast milk jaundice is a benign and self-limited condition, and interruption of breast feeding is not recommended as a therapeutic intervention' (37%). Table 2 presents scores for knowledge about neonatal jaundice among participating mothers.

Mothers' attitudes towards neonatal jaundice

The results revealed that 42.2% of participating mothers had poor attitudes towards neonatal jaundice. Over half of the participants strongly agreed that post-discharge observation was necessary and post-partum visits were needed to measure the bilirubin level (52.4% and 51.9%, respectively). However, some mothers believed that neonatal jaundice was a common physiological phenomenon that would not cause serious consequences (10.9%), and 29.2% lacked understanding that adequate feeding was conducive to resolving jaundice. Mothers' attitudes towards neonatal jaundice are presented in Table 3.

Mothers' practices regarding neonatal jaundice

In general, 53.1% of mothers had good practices regarding neonatal jaundice, and 96% checked their baby for

For peer review only

jaundice after discharge. However, only 41.9% indicated they would take the initiative to learn about neonatal jaundice after discharge. Mothers' practices regarding jaundice are shown in Table 4.

Factors associated with knowledge, attitudes and practices related to neonatal jaundice among mothers

The binary logistic regression analysis revealed that good knowledge about jaundice was associated with a high level of education (master's degree or above; odds ratio [*OR*]=5.977, 95% confidence interval [*CI*]: 1.994~17.916, *P*=0.001), receiving prior health education on neonatal jaundice (*OR*=3.617, 95%*CI*: 1.637~7.990, *P*=0.001), and male babies (*OR*=1.714, 95%*CI*: 1.122~2.167, *P*=0.013). A positive attitude towards jaundice was associated with being cared for by a '*yuesao*' (matron specialised in maternal and newborn care) (*OR*=1.969, 95%*CI*: 1.264~3.066, *P*=0.003) and good knowledge about jaundice (*OR*=1.804, 95%*CI*: 1.194~2.726, *P*=0.005). Finally, good practices related to jaundice were associated with prior health education on neonatal jaundice (*OR*=2.260, 95%*CI*: 1.105~4.625, *P*=0.026) and good knowledge about jaundice (*OR*=3.112, 95%*CI*: 2.040~4.749, *P*<0.001). The results of chi-square tests (bivariable analyses) for maternal knowledge, attitudes and practices related to neonatal jaundice are shown in supplementary table 1. Factors associated with knowledge, attitudes and practices related to neonatal jaundice among mothers are shown in Table 5.

For peer review only

DISCUSSION

In this study, 45.4% of mothers had good knowledge about neonatal jaundice. This knowledge level was better than that reported in other countries such as Nepal[23] (22%), Egypt [24] (30%) and Karbala city, Iraq[25] (34%). This may be attributed to the high proportion (83.1%) of our respondents who had graduated from university. Our study also found that education level was significantly associated with knowledge about neonatal jaundice, which was consistent with the results of an earlier review.[26] Due to differences in scoring algorithms or items investigating attitudes and practices[23–25], it was difficult to compare attitudes and behaviours reported in our study with those from other countries.

The majority (80.6%) of participants in this study had received prior health education on neonatal jaundice, which suggested that their knowledge about neonatal jaundice would be high. However, this study found that many mothers had poor knowledge regarding neonatal jaundice, with only 45.5% of participating mothers had good knowledge about neonatal jaundice. This large gap may be attributable to the gap of nearly a week between the time they received health education and the time of our investigation; some mothers might have forgotten the content of the health education. In addition, medical staff only provided post-discharge monitoring and follow-up instruction and did not include neonatal jaundice knowledge related to the questionnaire when conducting health education. Another factor that might have contributed to the comparative ineffectiveness of postnatal instruction was that the unique environment was absent that mothers received health education about jaundice from health workers in a single setting, which combined a lecture, demonstration and interactive discussion, as there is generally a rush to discharge mothers from birthing centres.

The present study reported that many mothers had poor knowledge regarding the causes and danger signs of jaundice. Relatively few mothers provided correct answers to some items; for example, 'Cranial haematoma may cause neonatal jaundice' (29%), 'Blood test is the gold standard for diagnosing neonatal jaundice' (34.2%), 'It is abnormal for jaundice to appear within 24 hours after birth' (36.2%), and 'It is abnormal for jaundice to reappear after it subsides' (37%). Poor knowledge about the causes and danger signs of jaundice may mean mothers turn to traditional treatments, which results in delays in seeking medical attention for neonatal jaundice, thereby contributing to the development of ABE and kernicterus.[11] Therefore, it is recommended that medical staff incorporate information about the causes

For peer review only

and danger signs of jaundice into neonatal jaundice health education programmes. For breast milk jaundice, this involves monitoring the jaundice without changing in the infant's breastfeeding;[27] however, 73% of mothers in this study did not know that breast milk jaundice is generally a benign condition, where interruption of breast feeding is not recommended as a therapeutic intervention. Having poor knowledge of breast milk jaundice may mean mothers discontinue breastfeeding after jaundice occurs. However brief, such discontinuation may jeopardise an infant's ability to return to exclusive breastfeeding, which is unnecessarily harmful to the infant and traumatic for mothers.[28] This means mothers of affected infants should be educated about breast milk jaundice and informed that breastfeeding should be interrupted in rare instances (i.e. if the neonate displays signs of ABE). In addition, the incidence of G6PD deficiency is high in Shenzhen.[29–30] Traditional Chinese medicine is widely used in China to prevent or treat neonatal jaundice.[31–32] However, neonates with G6PD deficiency that use such remedies may have severe jaundice.[33] Our findings suggested that most mothers (59.5%) did not know the G6PD deficiency may cause jaundice. Therefore, mothers whose neonates have G6PD deficiency should be educated about avoiding using traditional Chinese medicine to treat or prevent neonatal jaundice.

Importantly, our study highlighted that maternal information seeking behaviour related to neonatal jaundice needs to be improved. Only 41.7% of mothers in this study indicated they took the initiative to seek information about neonatal jaundice. This poor practice could be because physical and psychological changes in the postpartum period mean mothers' energy is limited. However, it could also be attributable to health literacy, which has an impact on people's health information seeking behaviour.[34] A previous study[34] reported that the lower the parents' health literacy, the less likely they were to take the initiative to obtain information about their child's health. Actively understanding relevant knowledge will help to improve maternal awareness of neonatal jaundice, which will be conducive to managing neonatal jaundice after discharge from hospital. In addition, with the popularisation of the Internet and smart phones, more parents are using these ways to access parenting knowledge. However, they complain that they face major challenges in identifying whether the information is reliable.[35-36] Therefore, it is recommended that when providing education about neonatal jaundice, medical staff also provide mothers with some reliable websites to facilitate the active information seeking about neonatal jaundice.

Our multivariate analysis revealed that mothers who had a male infant were more likely be

For peer review only

knowledgeable about neonatal jaundice compared with mothers who had a female infant. Health workers generally perform routine jaundice evaluation during birth hospitalisation, and male sex is a risk factor for neonatal jaundice.[37] We speculated that mothers who gave birth to male infants had more opportunities to receive information about neonatal jaundice. Having received prior education on neonatal jaundice from medical staff and a high education level were both determinants of knowledge about neonatal jaundice. This finding was consistent with the results of a study from Egypt[13] that found mothers who were university graduates had the highest knowledge scores. This suggested it is necessary for medical staff to provide information about jaundice to mothers with lower education levels.

Our multivariate analysis of attitudes revealed that mothers who were cared for by a *yuesao* were more likely to have positive attitudes toward neonatal jaundice than other mothers. Chinese tradition dictates that new mothers stay home and rest for a 'confinement period' of about 1 month (28–42 days) after giving birth, which is thought to facilitate recovery. Previous studies reported that being cared for by a *yuesao* during this stage can help improve the health of mothers and babies,[38-39] reduce postpartum depression,[40-41] and facilitate breastfeeding.[42] Therefore, hiring a *yuesao* to prepare post-partum foods and help with household and childcare tasks has become increasingly popular in many urban families. Our study showed that mothers who were cared for by a *yuesao* were more likely to respond positively to neonatal jaundice. This may be because the *yuesao* offers a valuable resource for jaundice counselling. Therefore, it is important that institutions that train *yuesao* should cover dealing with common problems such as neonatal jaundice as well as maternal and childcare skills.

Our multivariate analysis of practices related to neonatal jaundice revealed that mothers who had received prior education on neonatal jaundice from medical staff were more likely to have good practices related to jaundice than other mothers. This finding was consistent with a study conducted in Nigeria[43] that showed mothers who obtained knowledge about neonatal jaundice from medical staff were significantly less likely to self-treat and more likely to seek medical treatment promptly. We also found that mothers with good knowledge about neonatal jaundice were more likely to have good attitudes and practices; this was consistent with the 'knowledge, attitudes practices' (KAP) model,[44] which suggests greater knowledge is the basis for good attitudes and practices.

For peer review only

LIMITATIONS

This study had some limitations. One limitation was that when recruiting mothers, we might have motivated them to learn about neonatal jaundice, which would have improved mothers' awareness of neonatal jaundice before the investigation. However, Chinese traditional culture indicates that mothers need to confine themselves for 1 month after giving birth, so they do not leave their homes during this period. This also made it difficult to recruit mothers after discharge from the hospital. To increase the accessibility of the population, this study recruited mothers in advance during the hospitalisation period after delivery. Another limitation was that our research findings are only representative of mothers' KAP, but for some infants, the main caregivers are other people such as grandmothers, sisters or aunts. The findings of this study are not representative of these individuals.

CONCLUSION

Overall, mothers' knowledge about jaundice was low, especially regarding causes, danger signs and breast milk jaundice. Active information seeking behaviour about neonatal jaundice needs to be improved. Therefore, it is recommended that medical staff incorporate information about the causes, danger signs and breast milk jaundice into neonatal jaundice health education programmes and provide reliable websites for mothers to obtain information about neonatal jaundice. This study also showed that the mother's education level was an important factor that is significantly associated with knowledge about jaundice. In addition, mothers receiving care from a *yuesao* tend to have positive attitudes toward jaundice. Enhancing jaundice-related education programmes targeting mothers with a low education level and no *yuesao* care is important.

Acknowledgments: We would like to acknowledge seven neonatologists and my research team for giving feedback to the questionnaire development. Our appreciation also goes to the study participants for generously spending time and providing information in this survey.

Contributors: Y.H. and L.C. are joint first authors. Y.H., L.C., and X.J.W. designed the study. Y.H., X.J.W., C.Z., Z.L.G., F.Y., and J.L. collected the data. Y.H., L.C., and X.J.W. analyzed the data. HY drafted the manuscript. W.Z.C., Y.H. and L.C. contributed to the interpretation of the results and critical revision of the manuscript for important intellectual content and approved the final version of the manuscript. All authors have read and approved the final manuscript. W.Z.C.,Y.H.,and L.C.are the study

For peer review only

guarantors.

Funding: This study was supported by grants from Sanming Project of Medicine in ShenZhen, China (SZSM201612018).

Competing interests: None declared.

Patient consent for publication: Not aplicable.

Data availability statement: All data relevant to the study are included in the article or uploaded as supplementary information.

REFERENCES

1. Wang WP. Pediatrics. Poeple's Medical Publishing House. 2018.

2. Management of hyperbilirubinemia in the newborn infant 35 or more weeks of gestation. Pediatrics 2004;114(1):297-316.

3. Riordan SM, Shapiro SM. Review of bilirubin neurotoxicity I: molecular biology and neuropathology of disease. Pediatr Res 2020;87(2):327-31.

4. Karimzadeh P, Fallahi M, Kazemian M, et al. Bilirubin Induced Encephalopathy. Iran J Child Neurol. 2020;14(1):7-19.

5. Ma XL, Shi LP, Du LZ, et al. Clinical characteristics of bilirubin encephalopathy in Chinese newborn infants-a national multicenter survey. Zhonghua Er Ke Za Zhi 2012;50(05): 331-335.

6. Bhutani VK, Zipursky A, Blencowe H, et al. Neonatal hyperbilirubinemia and Rhesus disease of the newborn:incidence and impairment estimates for 2010 at regional and global levels. PEDIATRRES 2013;74:86-100.

7. Wennberg RP, Oguche S, Imam Z, et al. Maternal Instruction About Jaundice and the Incidenceof Acute Bilirubin Encephalopathy in Nigeria. J Pediatr 2020;221:47-54.

8. Dlala UM, Wennberg RP, Abdulkair I, et al. Patterns of acute bilirubin encephalopathy in Nigeria: a multicenter pre-intervention study. J Perinatol 2018;38(7):873-880.

9. Addendum to Jaundice in newborn babies under 28 days. London: National Institute for Health and Care Excellence (UK). 2016.

10. Watchko JF. Maternal Instruction on Neonatal Jaundice: What Can we Learn from the Stop Kernicterus in Nigeria (SKIN) Experience? J Pediatr 2020;221:7-8.

For peer review only

11. Salia SM, Afaya A, Wuni A, et al. Knowledge, attitudes and practices regarding neonatal jaundice among caregivers in a tertiary health facility in Ghana. PloS one 2021;16(6).

12. Amegan-aho KH, Segbefia CI, Glover N, et al. Neonatal Jaundice: awareness, perception and preventive practices in expectant mothers. Ghana Med J 2019;53(4):267-272.

13. Moawad EMI, Abdallah EAA, Ali YZA. Perceptions, practices, and traditional beliefs related to neonatal jaundice among Egyptian mothers. Medicine 2016;95(36):e4804.

14. Editorial Board of Chinese Journal of Pediatrics Subspecialty Group of Neonatology, The Society of Pediatrics, Chinese Medical Association. [Experts consensus on principles for diagnosis and treatment of neonatal jaundice]. Zhonghua Er Ke Za Zhi 2010;48:685–686.

15. Adoba P, Ephraim RKD, Kontor KA, et al. Knowledge Level and Determinants of Neonatal Jaundice: A Cross-Sectional Study in the Effutu Municipality of Ghana. Int J Pediatr 2018.

16. Demis A, Getie A, Wondmieneh A ,et al. Knowledge on neonatal jaundice and its associated factors among mothers in northern Ethiopia: a facility-based cross-sectional study. BMJ Open 2021;11(3):e44390.

17. Shi JC, Mo XK, Sun ZQ. Content validity index in scale development. Zhong Nan Da Xue Xue Bao Yi Xue Ban 2012;37(02):49-52.

18. Mcneish D. Thanks coefficient alpha, we'll take it from here. Psychological Methods 2018;23(3):412-433.

19. Li Z. Nursing Research Methods[M]. Poeple's Medical Publishing House. 2018.

20. Cronbach LJ. Coefficient alpha and the internal structure of tests. Psychometrika 1951;16(3):297-334.

21. Clark LAWD. Constructing validity: Basic issues in objective scale development. Psychol Assess 1995;7(3):309-19.

22. Li TT, Wang J, Chen X, Chen L, et al.Obstetric Nurses' Knowledge, Attitudes, and Professional Support Related to Actual Care Practices About Urinary Incontinence. Female Pelvic Med Reconstr Surg 2021;27(2):e377-384.

23. Shrestha S, Maharjan S, Petrini M. Knowledge about neonatal jaundice among Nepalese mothers. JBPKIHS 2019;2:34-42.

For peer review only

24. Kasemy ZA, Bahbah WA, El Hefnawy SM, et al. Prevalence of and mothers' knowledge, attitude and practice towards glucose-6-phosphate dehydrogenase deficiency among neonates with jaundice: a cross-sectional study. BMJ Open 2020;10(2): e34079.

25. Hussein H, Aziz A. Assessment of mothers' knowledge and beliefs toward care of neonatal jaundice in pediatric teaching hospital in Holy Karbala City. Int J Sci Res 2016;6.

26. Wennberg RP, Watchko JF, Shapiro SM. Maternal Empowerment - An Underutilized Strategy to Prevent Kernicterus? Curr Pediatr Rev 2017;13(3):210-219.

27. Bratton S, Cantu RM, Stern M, et al. Breast Milk Jaundice(Nursing). 2022.

28. Preer GL, Philipp BL. Understanding and managing breast milk jaundice. Arch Dis Child Fetal Neonatal Ed 2011;96(6):F461-6.

29. Gao J, Lin S, Chen S, et al. Molecular Characterization of Glucose-6-Phosphate Dehydrogenase Deficiency in the Shenzhen Population. Hum Hered 2020;85(3-6):110-116.

30. Liu Z, Yu C, Li Q, et al. Chinese newborn screening for the incidence of G6PD deficiency and variant of G6PD gene from 2013 to 2017. Hum Mutat 2020;41(1):212-221.

31. Standardization Project Team of Guidelines for Clinical Application of Chinese Patent Medicines for the Treatment of Predominant Diseases. Guidelines for clinical application of Chinese patentmedicine in the treatment of neonatal jaundice (2020). Chin J Integr Tradit Chin West Med 2021;41(03):280-285.

32. Gu Minyong, Yang Yan, Sun Yanli, et al. Guidelines for Clinical Diagnosis and Treatment ofPediatrics in Traditional Chinese Medicine · Neonatal jaundice (Revised). Pediatrics Journal of Traditional Chinese Medicine 2018;14(02):5-9.

33. Fok TF. Neonatal jaundice--traditional Chinese medicine approach. *J Perinatol*, 2001;21 Suppl 1: S98-S100, S104-S107.

34. Sørensen K, Van den Broucke S, Fullam J, et al. Health literacy and public health: a systematic review and integration of definitions and models. BMC Public Health 2012;12:80.

35. Kubb C, Foran HM. Online Health Information Seeking by Parents for Their Children: Systematic Review and Agenda for Further Research. J Med Internet Res 2020;22(8):e19985.

For peer review only

36. Smith CA, Parton C, King M, et al. Parents' experiences of information-seeking and decision-making regarding complementary medicine for children with autism spectrum disorder: a qualitative study. BMC Complement Med Ther 2020;20(1):4.

37. Birhanu MY, Workineh AA, Molla Y, et al. Rate and Predictors of Neonatal Jaundice in Northwest Ethiopia: Prospective Cohort Study. J Multidiscip Healthc 2021;14:447-457.

38. Li YY, Wang HY. The impact of yuesao on the health of mothers and infants. Chinese JounalOf Practical Actical Nursing 2010;26(27): 42-43.

39. Yang L, Zhu MY, Yin Y. Impact of babysitter's care on health status of infants: a survey study. Journal of Nursing Science 2011;(18):87-88.

40. Xing CG. Practical Strategies and Policy Implications of Postpartum Doulas' Intervention on Postpartum Depression. Medicine and Society 2021;34(04):11-16.

41. Peng K, Zhou L, Liu X, et al. Who is the main caregiver of the mother during the doing-the-month: is there an association with postpartum depression? BMC Psychiatry 2021;21(1):270.

42. Wu W, Zhang J, Silva ZI, et al. Factors influencing breastfeeding practices in China: A meta-aggregationof qualitative studies. Matern Child Nutr 2021:e13251.

43. Ezeaka CV, Ugwu RO, Mukhtar-Yola M, et al. Pattern and predictors of maternal care-seekingpractices for severe neonatal jaundice in Nigeria: a multi-centre survey. BMC Health Serv Res 2014;14:192.

44. Badran IG. Knowledge, attitude and practice the three pillars of excellence and wisdom: a place in themedical profession. 1995.

Figure legend

Figure 1 Data collection procedures

For peer review only

Table 1. Baseline characteristics of participating mothers (N=403)

Variables	Characteristics	n (%)
**Sociodemographic data**		
Age, years	19~27	114 (28.3)
	28~32	197 (48.9)
	33~45	92 (22.8)
Blood group	O	145 (36.0)
	A	121 (30.0)
	B	106 (26.3)
	AB	31 (7.7)
Education level	High school and below	68 (16.9)
	University	310 (76.9)
	Postgraduate and above	25 (6.2)
Occupation	Employed	267 (66.3)
	Self-employed	40 (9.9)
	Homemaker	82 (20.3)
	Others	14 (3.5)
Average family monthly income, RMB	≤5000	50 (12.4)
	5001~10000	154 (38.2)
	10001~20000	125 (31)
	20001~30000	35 (8.7)
	≥30001	39 (9.7)
Time from the place of residence to the delivery hospital, minutes	≤10	52 (12.9)
	10~30	212 (52.6)
	30~60	126 (31.3)
	≥60	13 (3.2)
**Delivery history**		
Parity	Primipara	224 (55.6)
	Multipara	179 (44.4)
Delivery mode	Spontaneous vaginal	305 (75.7)
	Caesarean section	98 (24.3)
**Infant's Information**		
Sex	Male	210 (52.1)
	Female	193 (47.9)

For peer review only

Birth weight[1]	Low	365 (7.7)
	Normal	31 (90.6)
	Hight	7 (1.7)
Feeding method	Exclusive breastfeeding	196 (48.6)
	Mixed feeding	197 (48.9)
	Exclusive formula-feeding	10 (2.5)
Cranial haematoma[2]	Yes	15 (3.7)
	No	364 (90.3)
	Not sure	24 (6.0)
Whether meconium passed within 24 hours	Yes	397 (98.5)
	No	6 (1.5)
Predischarge bilirubin level	Normal	312 (77.4)
	Height	91 (22.6)
'Yuesao'[3]	Yes	138 (34.2)
	No	265 (65.8)
**Previous experience/exposure to neonatal jaundice**		
Prior health education on neonatal jaundice	Yes	361 (80.6)
	No	42 (10.4)
Family history/friends with neonatal jaundice history(N=373)	Yes	45 (12.1)
	No	328 (87.9)
Previous child with a history of neonatal jaundice	Yes	56 (13.9)
	No	347 (86.1)
Current child admitted to hospital for treatment for jaundice after discharge	Yes	113 (28.0)
	No	290 (72.0)
Mother's knowledge level	Good	183 (45.4)
	Poor	220 (54.6)
Mother's attitude level	Good	170 (42.2)
	Poor	233 (57.8)
Mother's practice level	Good	214 (53.1)
	Poor	183 (46.9)

Note: [1.]Weight: low weight <2500 g; normal weight 2500–4000 g; high weight >4000 g. [2.]Cranial haematoma: haematoma caused by rupture and bleeding of subperiosteal v essels in the parieto-occipital region due to birth injury. [3.]*yuesao*: maternity matron sp ecialised in caring for mothers and newborns.

For peer review only

Table 2. Maternal knowledge about neonatal jaundice (N=403)

Items	True n (%)	False n (%)	Don't know n (%)	Correct rate n (%)
**Observation of neonatal jaundice**				
When newborns develop jaundice, their skin will turn yellow.	390 (96.8)	4 (1.0)	9 (2.2)	390 (96.8)
When newborns develop jaundice, their face will turn yellow first.	344 (85.4)	12 (3.0)	47 (11.7)	344 (85.4)
When looking for jaundice, check the naked baby in bright and preferably natural light.	365 (90.6)	10 (2.5)	28 (6.9)	365 (90.6)
**Classification of neonatal jaundice**				
Neonatal jaundice is divided into physiological jaundice and pathological jaundice.	371 (92.1)	3 (0.7)	29 (7.2)	371 (92.1)
**Danger signs of neonatal jaundice**				
Palms and soles turn yellow, indicating that jaundice is severe.	265 (65.8)	29 (7.2)	109 (27.0)	265 (65.8)
It is an abnormal condition if the jaundice appears within first 24 h.	146 (36.2)	155 (38.5)	102 (25.3)	146 (36.2)
It is an abnormal condition that the jaundice reappears after it has subsided.	149 (37.0)	143 (35.5)	111 (27.5)	149 (37.0)
**Complication of neonatal jaundice**				
Severe jaundice may lead to brain damage.	325 (80.6)	7 (1.7)	71 (17.6)	325 (80.6)
**Cause of neonatal jaundice**				
The mother's blood type is O, and the father's blood type is A, B, or AB, which may cause neonatal jaundice.	228 (56.6)	38 (9.4)	137 (34.0)	228 (56.6)
Cranial hematoma may cause neonatal jaundice.	117 (29.0)	39 (9.7)	247 (61.3)	117 (29.0)
Bowel obstruction may cause neonatal jaundice.	281 (69.7)	14 (3.5)	108 (26.8)	281 (69.7)
Broad bean disease (G6PD) may cause jaundice.	163 (40.4)	25 (6.2)	215 (53.3)	163 (40.4)

For peer review only

Breastfeeding may cause jaundice	200 (49.6)	109 (27.0)	94 (23.3)	200 (49.6)
**Breast milk jaundice**				
Breast milk jaundice is a benign and self-limited condition, and interruption of breast feeding is not recommended as a therapeutic intervention.	149 (37.0)	135 (33.5)	119 (29.5)	149 (37.0)
**Diagnosis of neonatal jaundice**				
Blood test is the gold standard for diagnosing neonatal jaundice.	138 (34.2)	127 (31.5)	138 (34.2)	138 (34.2)
**Treatment of neonatal jaundice**				
Phototherapy is a common, effective and safe treatment method for neonatal jaundice.	367 (91.1)	3 (0.7)	33 (8.2)	367 (91.1)

Table 3. Maternal attitudes towards neonatal jaundice (N=403)

Items	Strongly disagree n (%)	Disagree n (%)	Not sure n (%)	Agree n (%)	Strongly agree n (%)
I think neonatal jaundice is a common physiological phenomenon and will not cause serious consequences. (-)	80 (19.1)	216 (53.6)	63 (15.6)	39 (9.7)	5 (1.2)
I think that a baby with jaundice, does not need treatment and will self-recover. (-)	151 (38.5)	201 (49.9)	43 (10.7)	6 (1.5)	2 (0.5)
I think it is very important to observe neonatal jaundice after discharged from the hospital.	5 (1.2)	0 (0.0)	6 (1.5)	181 (44.9)	211 (52.4)
I think it is necessary for post-partum visitors to assess jaundice condition.	8 (1.0)	0 (0.0)	8 (2.0)	182 (45.2)	209 (51.9)
I think a baby with suspected jaundice should go to a medical institution or community healthcare centre to measure the bilirubin level	3 (0.7)	5 (1.2)	10 (2.5)	230 (57.1)	155 (37.5)

For peer review only

in a timely manner.					
I believe that adequate breastfeeding is good for jaundice.	5 (1.2)	29 (7.2)	84 (20.8)	196 (48.6)	89 (22.1)
I think it is necessary to learn knowledge of neonatal jaundice.	2 (0.5)	0 (0.0)	10 (2.5)	216 (53.6)	175 (43.4)

Note: - represents reverse scored items.

Table 4. Maternal practices regarding neonatal jaundice (N=403)

**Items**	**Yes** n (%)	**No** n (%)
I took the initiative to seek information about neonatal jaundice.	168 (41.7)	235 (58.3)
After discharge, I checked my infant for jaundice, such as the colour of the skin, sclerae, urine, bowel movements.	387 (96.0)	16 (4.0)
After discharge, I followed the doctor's instructions to take the infant to a medical institution or community healthcare centre to measure the bilirubin level.	376 (93.3)	27 (6.7)
After discharge, I breastfed adequately.	359 (89.1)	44 (10.9)

For peer review only

Table 5. Binary logistic regression analysis of maternal knowledge, attitudes and practices related to neonatal jaundice (N=403)

Variables	Classification	Knowledge			Attitude			Behaviour		
		OR	*95%CI*	*P*	*OR*	*95%CI*	*P*	*OR*	*95%CI*	*P*
Education level	High school and below (Ref.)			**0.001**						
	College and undergraduate course	3.011	1.563~5.800	0.001		*NI*			*NI*	
	Postgraduate student or above	5.977	1.994~17.916	0.001						
Occupation	Employed (Ref.)			0.110						
	Self-employed	0.403	0.183~0.887	0.024		*NI*			*NI*	
	Homemaker	0.730	0.409~1.302	0.286						
	Other	1.266	0.401~3.994	0.688						
Parts	Primipara (Ref.)		*NI*						*NI*	
	Multipara				1.498	0.995~2.255	0.053			
Prior education on neonatal jaundice	No (Ref.)					*NI*				
	Yes	3.617	1.637~7.993	**0.001**				2.260	1.105~4.625	**0.026**
Neonate sex	Female (Ref.)					*NI*			*NI*	
	Male	1.714	1.122~2.617	**0.013**						
Cranial hematoma	No (Ref.)			0.073						
	Yes	1.549	0.510~4.706	0.440		*NI*			*NI*	
	It is not clear	0.321	0.112~0.920	0.034						
Yuesao	No (Ref.)		*NI*						*NI*	

For peer review only

	yes		1.969	1.264~3.066	**0.003**			
Knowledge level	Poor (Ref.)	*NA*		*NI*				
	good		1.804	1.194~2.726	**0.005**	3.112	2.040~4.749	**0.000**
Attitude level	poor (Ref.)	*NI*		*NA*				
	good					1.498	0.983~2.283	0.060

Note: *OR,* odds ratio; *CI* confidence interval; *NI*, not included in the final logistic regression analysis; *NA*, not applicable.

For peer review only

For peer review only

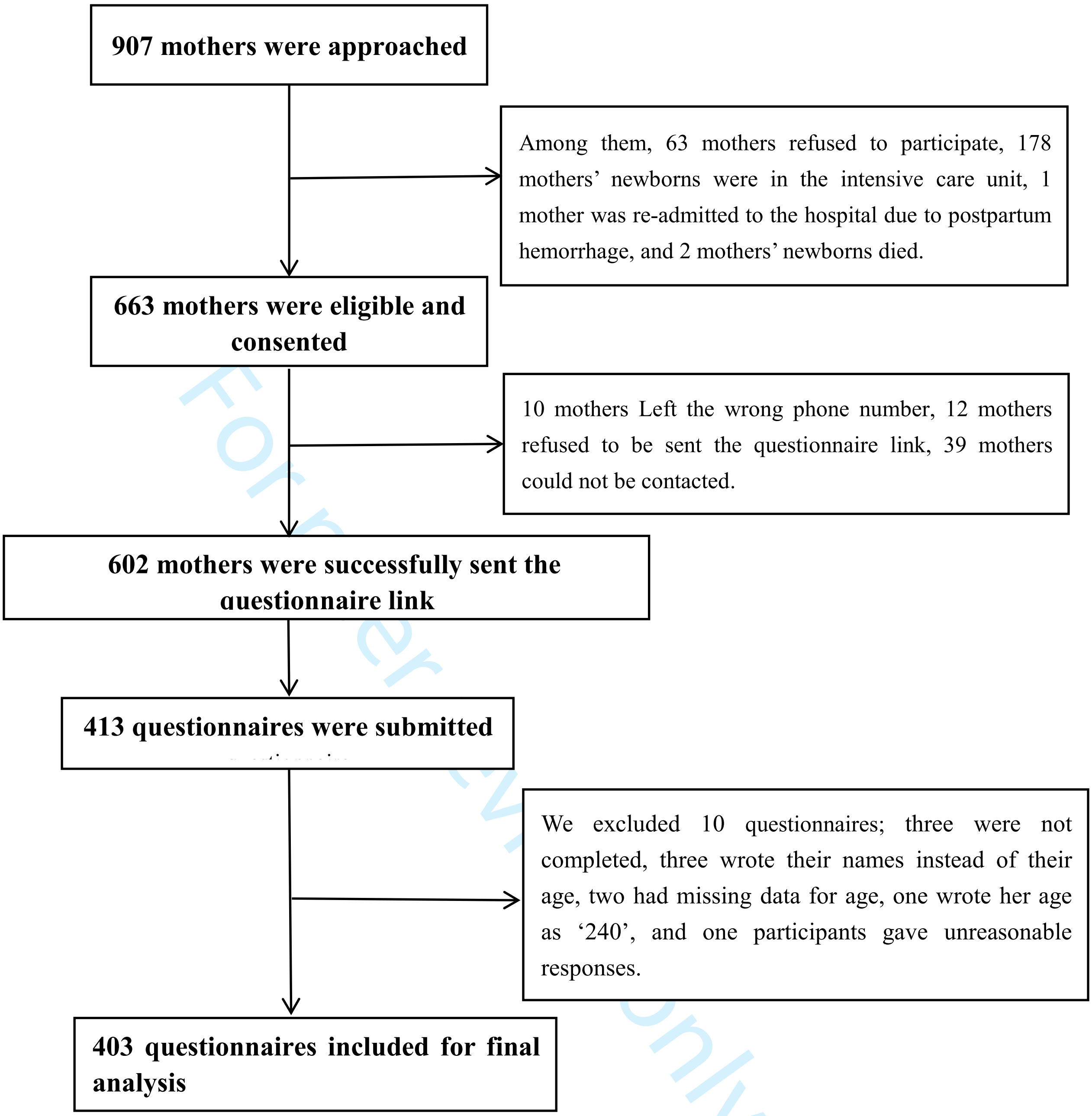
907 mothers were approached
Among them, 63 mothers refused to participate, 178 mothers' newborns were in the intensive care unit, 1 mother was re-admitted to the hospital due to postpartum hemorrhage, and 2 mothers' newborns died.
663 mothers were eligible and consented
10 mothers Left the wrong phone number, 12 mothers refused to be sent the questionnaire link, 39 mothers could not be contacted.
602 mothers were successfully sent the questionnaire link
413 questionnaires were submitted
We excluded 10 questionnaires; three were not completed, three wrote their names instead of their age, two had missing data for age, one wrote her age as '240', and one participants gave unreasonable responses.
403 questionnaires included for final analysis

Supplementary table 1. Chi-square analyses of maternal knowledge, attitudes and practices related to neonatal jaundice (N=403)

Variables		Knowledge*		Attitude*		Behaviour*	
		χ^2	*p*	χ^2	*p*	χ^2	*p*
**Sociodemographic data**							
Age(years)	19~27<br>28~32<br>33~45	2.008	0.366	1.385	0.500	0.292	0.864
Blood group	O<br>A<br>B<br>AB	1.668	0.644	3.873	0.276	4.244	0.236
Education level	High school and below<br>University<br>Postgraduate and above	**16.921**	**0.000**	**9.800**	**0.007**	**6.084**	**0.048**
Occupation	Employed<br>Self-employed<br>Housewife<br>Others	**15.279**	**0.002**	2.007	0.571	6.865	0.076
Average family monthly income (RMB)	≤5000<br>5001~10000<br>10001~20000<br>20001~30000<br>≥30001	**10.444**	**0.034**	6.849	0.144	8.683	0.070
Time from the place of residence to the delivery hospital (minutes)	≤10<br>10~30<br>30~60<br>≥60	1.458	0.692	3.289	0.349	1.635	0.652
**Delivery history**							
Parity	Primiparous<br>Multipara	0.021	0.885	**4.536**	**0.033**	0.629	0.428
Delivery mode	Spontaneous vaginal<br>Caesarean section	0.014	0.907	0.152	0.696	0.500	0.479
**Infant's Information**							
Sex	Male<br>Female	**6.409**	**0.011**	2.241	0.134	0.804	0.370

For peer review only

Birth weight[1]	Low<br>Normal<br>Hight	1.008	0.615	2.034	0.402	4.216	0.130
Feeding way	Exclusive breastfeeding<br>Mixed feeding<br>Exclusive Formula-feeding	5.479	0.065	0.873	0.647	5.604	0.062
Cranial hematoma[2]	Yes<br>No<br>Not sure	**7.288**	**0.026**	0.342	0.843	0.380	0.827
Whether to pass meconium with in 24 hours	Yes<br>No	0.052	0.820	1.626	0.202	3.247	0.072
Predischarge bilirubin level	Normal<br>Hight	1.253	0.263	0.022	0.882	1.837	0.175
"yuesao" [3]	Yes<br>No	1.067	0.320	**11.878**	**0.001**	0.327	0.567
**Previous experience/exposure to NNJ**							
Prior health education on neonatal jaundice	Yes<br>No	**8.824**	**0.003**	0.179	0.672	**9.236**	**0.002**
Previous child history of neonataljaundice	Yes<br>No	1.067	0.302	0.140	0.708	0.133	0.715
Current child admitted to the hospital for treatment due to jaundice after disch arge	Yes<br>No	0.358	0.549	0.012	0.912	3.156	0.076
Family history/friends with NNJ history（N=373)	Yes<br>No	0.351	0.554	0.100	0.752	0.860	0.354
Mother's knowledge level	good<br>poor	*NA*		**9.478**	**0.002**	**35.751**	**0.000**
Mother's attitude level	good<br>poor	*NA*		*NA*		**7.179**	**0.007**

Note: *NA*, not applicable. *Knowledge attitude and behaviour were all divided into two levels (po or or good)

For peer review only

STROBE Statement—Checklist of items that should be included in reports of ***cross-sectional studies***

	Item No	Recommendation	Reported on page number
**Title and abstract**	1	(*a*) Indicate the study's design with a commonly used term in the title or the abstract	Page 1:title page
		(*b*) Provide in the abstract an informative and balanced summary of what was done and what was found	Page 2-3
**Introduction**			
Background/rationale	2	Explain the scientific background and rationale for the investigation being reported	Page 3-4
Objectives	3	State specific objectives, including any prespecified hypotheses	Page 4
**Methods**			
Study design	4	Present key elements of study design early in the paper	Page 4
Setting	5	Describe the setting, locations, and relevant dates, including periods of recruitment, exposure, follow-up, and data collection	Page 4 and Figure 1data collection progress
Participants	6	(*a*) Give the eligibility criteria, and the sources and methods of selection of participants	Page 4
Variables	7	Clearly define all outcomes, exposures, predictors, potential confounders, and effect modifiers. Give diagnostic criteria, if applicable	Page 6:The levels of knowledge, attitude and practice (for poor, for good)
Data sources/ measurement	8*	For each variable of interest, give sources of data and details of methods of assessment (measurement). Describe comparability of assessment methods if there is more than one group	Page 4-6
Bias	9	Describe any efforts to address potential sources of bias	Page 2-3 Strengths and limitations of this study
Study size	10	Explain how the study size was arrived at	NI:Sufficient sample size
Quantitative variables	11	Explain how quantitative variables were handled in the analyses. If applicable, describe which groupings were chosen and why	Page 6
Statistical methods	12	(*a*) Describe all statistical methods, including those used to control for confounding	Page 6
		(*b*) Describe any methods used to examine subgroups and interactions	Page 6
		(*c*) Explain how missing data were addressed	Page 5-6 excluded 10 participants' data
		(*d*) If applicable, describe analytical methods taking account of	NA

For peer review only - http://bmjopen.bmj.com/site/about/guidelines.xhtml

		sampling strategy	
		(*e*) Describe any sensitivity analyses	NA
**Results**			
Participants	13*	(a) Report numbers of individuals at each stage of study—eg numbers potentially eligible, examined for eligibility, confirmed eligible, included in the study, completing follow-up, and analysed	Figure 1data collection progres
		(b) Give reasons for non-participation at each stage	Figure 1data collection progres
		(c) Consider use of a flow diagram	Figure 1data collection progres
Descriptive data	14*	(a) Give characteristics of study participants (eg demographic, clinical, social) and information on exposures and potential confounders	Page 6 and Table1
		(b) Indicate number of participants with missing data for each variable of interest	NI
Outcome data	15*	Report numbers of outcome events or summary measures	Page 6-7
Main results	16	(*a*) Give unadjusted estimates and, if applicable, confounder-adjusted estimates and their precision (eg, 95% confidence interval). Make clear which confounders were adjusted for and why they were included	Page 7 and Table5
		(*b*) Report category boundaries when continuous variables were categorized	NI
		(*c*) If relevant, consider translating estimates of relative risk into absolute risk for a meaningful time period	NA
Other analyses	17	Report other analyses done—eg analyses of subgroups and interactions, and sensitivity analyses	NA
**Discussion**			
Key results	18	Summarise key results with reference to study objectives	Page 7-9
Limitations	19	Discuss limitations of the study, taking into account sources of potential bias or imprecision. Discuss both direction and magnitude of any potential bias	Page 2-3
Interpretation	20	Give a cautious overall interpretation of results considering objectives, limitations, multiplicity of analyses, results from similar studies, and other relevant evidence	Page 10
Generalisability	21	Discuss the generalisability (external validity) of the study results	Page 7-9
**Other information**			
Funding	22	Give the source of funding and the role of the funders for the present study and, if applicable, for the original study on which the present article is based	Page 10-11

Note: *NI*, Not included ; *NA*, Not available

For peer review only - http://bmjopen.bmj.com/site/about/guidelines.xhtml

BMJ Open

Maternal knowledge, attitudes and practices related to neonatal jaundice and associated factors in Shenzhen, China: a facility-based cross-sectional study.

Journal:	*BMJ Open*
Manuscript ID	bmjopen-2021-057981.R2
Article Type:	Original research
Date Submitted by the Author:	06-Jul-2022
Complete List of Authors:	Huang, Ying; Shenzhen Hospital of Southern Medical University, Department of Nursing; Southern Medical University, School of Nursing,<br>Chen, Ling; Shenzhen Hospital of Southern Medical University, Department of Nursing<br>Wang, Xiaojiao; Shenzhen Hospital of Southern Medical University, Department of Nursing<br>Zhao, Chun; Shenzhen Hospital of Southern Medical University, Department of Nursing<br>Guo, Zonglian; Shenzhen Hospital of Southern Medical University, Department of Obstetrics<br>Li, Jue; Shenzhen Hospital of Southern Medical University, Department of Nursing<br>Yang, Fang; shen zhen shi bao an qu fu you bao jian yuan, Department of Obstetrics<br>Cai, Wenzhi; Shenzhen Hospital of Southern Medical University, Department of Nursing; Southern Medical University, School of Nursing
**Primary Subject Heading**:	Health informatics
Secondary Subject Heading:	Health services research, Health informatics, Public health
Keywords:	NEONATOLOGY, MEDICAL EDUCATION & TRAINING, Child protection < PAEDIATRICS

SCHOLARONE™
Manuscripts

I, the Submitting Author has the right to grant and does grant on behalf of all authors of the Work (as defined in the below author licence), an exclusive licence and/or a non-exclusive licence for contributions from authors who are: i) UK Crown employees; ii) where BMJ has agreed a CC-BY licence shall apply, and/or iii) in accordance with the terms applicable for US Federal Government officers or employees acting as part of their official duties; on a worldwide, perpetual, irrevocable, royalty-free basis to BMJ Publishing Group Ltd ("BMJ") its licensees and where the relevant Journal is co-owned by BMJ to the co-owners of the Journal, to publish the Work in this journal and any other BMJ products and to exploit all rights, as set out in our licence.

The Submitting Author accepts and understands that any supply made under these terms is made by BMJ to the Submitting Author unless you are acting as an employee on behalf of your employer or a postgraduate student of an affiliated institution which is paying any applicable article publishing charge ("APC") for Open Access articles. Where the Submitting Author wishes to make the Work available on an Open Access basis (and intends to pay the relevant APC), the terms of reuse of such Open Access shall be governed by a Creative Commons licence – details of these licences and which Creative Commons licence will apply to this Work are set out in our licence referred to above.

Other than as permitted in any relevant BMJ Author's Self Archiving Policies, I confirm this Work has not been accepted for publication elsewhere, is not being considered for publication elsewhere and does not duplicate material already published. I confirm all authors consent to publication of this Work and authorise the granting of this licence.

For peer review only

Title: Maternal knowledge, attitudes and practices related to neonatal jaundice and associated factors in Shenzhen, China: a facility-based cross-sectional study

Basic information about the co-authors:

Ying Huang: 1. Shenzhen Hospital, Southern Medical University. 2. School of Nursing, Southern Medical University

Ling Chen: Shenzhen Hospital, Southern Medical University

Basic information about other authors:

XiaoJiao Wang: Shenzhen Hospital, Southern Medical University.

Chun Zhao: Shenzhen Hospital, Southern Medical University.

ZongLian Guo: Shenzhen Hospital, Southern Medical University.

Jue Li: Shenzhen Hospital, Southern Medical University.

Fang Yang: Shen zhen shi bao an qu fu you bao jian yuan

Corresponding author:

WenZhi Cai: 1. Shenzhen Hospital, Southern Medical University. 2. School of Nursing, Southern Medical University; Shenzhen Hospital, Southern Medical University, Xinhu Road 1333, Baoan District, 518101 Shenzhen, China; Email: caiwzh@smu.edu.cn; Tel: +86-18002575566

Key Words: Jaundice, Neonate; Mothers; Health Knowledge, Attitudes, Practice;

Word count (excluding title page, abstract, literature, drawings and tables): 4076

For peer review only

ABSTRACT

Objective: This study aimed to assess knowledge, attitudes and practices related to neonatal jaundice among mothers in Shenzhen, China and analyse associated factors.

Design: A cross-sectional study.

Setting: This study was conducted in Shenzhen Hospital, Southern Medical University, a university-affiliated, tertiary level-A, public hospital in China. On average, 4000 mothers are discharged from this hospital after childbirth each year, most of whom can access a mobile phone and the Internet.

Participants: Participants were 403 mothers discharged from the study hospital within 48 to 72 hours after vaginal delivery or 96 to120 hours after caesarean delivery between April and June 2021. Participants were recruited using convenience sampling.

Primary outcome: Mothers' knowledge, attitudes and practices related to neonatal jaundice, modelled using binary logistic regression.

Secondary outcomes: Factors associated with mothers' knowledge, attitudes and practices related to neonatal jaundice.

Results: The questionnaire was reliable (Cronbach's alpha=0.802) and valid (scale-level content validity index=0.958). The valid response rate was 96.4%. Only 46.4% of participating mothers had good knowledge about neonatal jaundice and 41.7% indicated they would seek information about neonatal jaundice. A binary logistic regression analysis showed good knowledge about jaundice was associated with a high education level (i.e., master's degree or above; odds ratio [*OR*]=5.977, 95% confidence interval [*CI*]: 1.994~17.916, *P*=0.001), prior education on neonatal jaundice (*OR*=3.617, 95%*CI*: 1.637~7.990, *P*=0.001), and male babies (*OR*=1.714, 95%*CI*: 1.122~2.167, *P*=0.013). A positive attitude toward jaundice was associated with being cared for by a '*yuesao*' (maternity matron specialised in caring for mothers and newborns) (*OR*=1.969, 95%*CI*: 1.264~3.066, *P*=0.003) and good knowledge about jaundice (*OR*=1.804, 95%*CI*: 1.194~2.726, *P*=0.005). Finally, good practices related to neonatal jaundice were associated with prior education on neonatal jaundice (*OR*=2.260, 95%*CI*: 1.105~4.625, *P*=0.026) and good knowledge about jaundice (*OR*=3.112, 95%*CI*: 2.040~4.749, *P*<0.001).

Conclusion: Many mothers have poor knowledge about jaundice, especially regarding causes, danger signs and breast milk jaundice. Maternal information seeking behaviour about neonatal jaundice needs to be improved. Medical staff should incorporate information about the causes/danger signs of jaundice and breast milk jaundice in maternal health education. It is also necessary to strengthen health education for mothers, especially those with low education and no yuesao, and provide reliable websites where mothers can obtain information about neonatal jaundice.

For peer review only

Strengths and limitations of this study

- A strength of this study was that participants were mothers with healthy infants who had been discharged from the birth hospital; these mothers may be more likely to ignore the problem of jaundice.
- The investigation time was the peak period of jaundice occurrence, which aimed to avoid recall bias.
- Recruiting mothers and then surveying them at a later time may have prompted mothers to search for information about jaundice.
- This study focused on new mothers and did not include significant others who may have roles in care of the newborn (e.g. grandmothers, sisters or aunts).

INTRODUCTION

Neonatal jaundice, also known as neonatal hyperbilirubinemia, refers to yellow staining of the skin or other organs caused by the accumulation of bilirubin in the body.[1] It is a common clinical problem in the neonatal period, and approximately 50%~60% of full-term infants and 80% of premature infants develop jaundice within 1 week after birth.[2] In many infants, neonatal jaundice is a benign condition. However, severe hyperbilirubinemia may cause acute bilirubin encephalopathy (ABE) or kernicterus, which may progress to nerve deafness, choreoathetoid cerebral palsy, intellectual disability and even death.[3-4] A report from China showed that from January to December 2009, 348 cases of kernicterus were recorded in 33 hospitals.[5] In addition, a worldwide survey reported that at least 480,700 newborns develop severe hyperbilirubinemia each year, with the risk for kernicterus being 13% (n=75,400) and that for death being 24% (n=114,100).[6] Therefore, neonatal jaundice is a serious threat to the life and health of newborns, and the associated high rates of disability and mortality place heavy burdens on society and families.

Early detection and timely treatment of neonatal jaundice are key strategies to prevent ABE and kernicterus. However, neonatal jaundice generally peaks on the 5th~7th day after birth,[1] at which time most healthy full-term infants have been discharged from hospital. Therefore, most neonatal jaundice occurs at home. As the main caregivers of newborns after discharge from hospital, mothers are often the first to observe jaundice, its progression, and early signs of ABE and kernicterus. They are central to achieving favourable outcomes for management of neonatal jaundice. Wennberg[7] et al reported that providing mothers with detailed information about neonatal jaundice and its risks was associated with a reduction in the incidence of ABE in Nigeria. The Stop Kernicterus in Nigeria (SKIN) consortium[8] demonstrated that a delay in seeking care, regardless of birth site, was a major contributor to ABE and kernicterus and proposed that maternal education on neonatal jaundice should be targeted as an intervention strategy. The clinical practice guidelines for neonatal jaundice from the National Institute for Health and Care Excellence[9] and American Academy of Pediatrics Subcommittee[2] also recommend

For peer review only

parents and caregivers are educated about neonatal jaundice, especially on how to check their baby for jaundice and what to do when jaundice is suspected. These guidelines suggest that maternal jaundice instruction be given high priority among the myriad topics. However, effective instruction starts with meaningful engagement between hospital staff and mothers.[10] Hospital staff therefore need to clarify what mothers know about jaundice and their current attitudes and practices, which will allow health education programmes to target identified gaps. Although similar investigations have been done in other countries or regions including Ghana,[11] Accra,[12] and Egypt,[13] no evaluation tools or reports related to maternal knowledge, attitudes and practices about neonatal jaundice are available in China.

Therefore, this study designed a questionnaire to assess knowledge, attitudes and practices related to neonatal jaundice among mothers in Shenzhen, China. The information obtained maybe used to assist healthcare providers in designing educational programmes to improve awareness about neonatal jaundice among mothers, which will ultimately help to prevent disability and deaths among newborns.

METHODS

Study setting and design

We conducted an anonymous, self-administered, cross-sectional survey involving 403 mothers in Shenzhen, China from April to June 2021. Participants were recruited using convenience sampling.

Study population

The target population was mothers who gave birth at Shenzhen Hospital, Southern Medical University, China from April to June 2021.

Inclusion and exclusion criteria

Mothers were eligible for this study if they were discharged from the hospital without serious illness after childbirth and had access to a mobile phone and the Internet. Mothers were excluded if they were not the main caregiver of their newborn after being discharged from hospital or could not complete the questionnaire by themselves. Moreover, we excluded mothers whose newborns were admitted to the neonatal intensive care unit for treatment or that died after birth.

Ethical considerations

Ethical clearance was obtained from the Ethical Review Committee of Shenzhen Hospital of Southern Medical University (approval number: NYSZYYEC20210004). Data were collected from each participant after they received a clear explanation of the purpose and importance of this study and provided informed consent. Participating mothers were informed that participation was voluntary, and they could withdraw from the study at any time or refuse to answer any question. They were also informed they could ask for clarification about any

For peer review only

aspect of the study and that the study would not cause harm. Participants did not receive any monetary incentive to participate in this study. All personal information was de-identified and kept securely, and every effort was made to maintain participants' confidentiality. Furthermore, after the investigation, each mother was informed via mobile phone text messages about seeking healthcare from a nearby clinic immediately if any signs of jaundice were identified.

Data collection tools

Data were collected using a questionnaire that covered baseline characteristics, knowledge, attitudes and practices regarding neonatal jaundice. These items were developed with reference to: 1) an established guideline on neonatal jaundice,[9] 2) an integrative review,[14] and 3) three services for investigating maternal knowledge, attitude and behaviour regarding neonatal jaundice.[13, 15, 16] We also consulted seven neonatologists and discussed the items among the research team. Following the review by the expert panel, nine mothers whose babies had experienced neonatal jaundice were conveniently recruited to provide input on the importance and clarity of the questionnaire items. Mothers were asked to suggest alternative wording for existing items and identify items that required deletion and addition as necessary. Some items were modified based on this review. For example, we modified 'Have you ever learned about neonatal jaundice' to 'Prior health education on neonatal jaundice', 'G6PD deficiency' to 'Broad bean disease', and 'Serum total bilirubin is the gold standard for diagnosing neonatal jaundice' to 'Blood test is the gold standard for diagnosing neonatal jaundice'. No items were identified for deletion or addition. The draft questionnaire was then pretested with 20 mothers who were conveniently selected from the same hospital based on the study inclusion criteria and exclusion criteria. The final version of the questionnaire included 27 items. Sixteen items evaluated knowledge (categorical responses: 'true', 'false', 'do not know'), seven items assessed attitude (categorical responses: 'strongly agree', 'agree', 'not sure', 'disagree', 'strongly disagree'), and four items covered practices (yes/no responses).

Validity and reliability

The content validity of the questionnaire was appraised quantitatively by sending the final version of the questionnaire to a group of experts including seven specialists in the field of neonatology. Based on the experts' feedback about relevance, the instrument's scale-level content validity index was calculated as 0.958, which was higher than the value of 0.8 that indicates adequate validity.[17]

Cronbach's alpha[18] was used to check the reliability of the questionnaire, which is the most common measure of internal consistency. In this study, the Cronbach's alphas were 0.802 for the whole questionnaire, 0.789 for the knowledge dimension, 0.721 for the attitude dimension and 0.414 for the practice dimension. The Cronbach's alphas for the knowledge and attitude dimensions were higher than 0.7, and were within the acceptable

For peer review only

recommended range. However, that for practice (0.414) was below 0.7.[19] The small number of items (four items) on that dimension may explain the low alpha estimate. To avoid the impact of a small number of items, Cronbach[20] proposed the mean inter-item correlation (ρ) in 1951 to estimate the internal consistency of dimensions with a small number of items. Generally, a mean inter-item correlation (ρ) between 0.15 and 0.20 indicates acceptable internal consistency. [21] In this study, the mean inter-item correlation was 0.15 for the practice dimension, which was within the acceptable range.

Data collection procedure

On the day each mother was discharged after delivery, the investigator informed them of the purpose, duration and method of this study, and obtained their phone number. About 5 days after discharge from hospital, the investigator sent the questionnaire link, which was developed using the 'Question star' platform, to participating mothers via mobile phone and then called the participant to complete the questionnaire on the same day. In total, 413 questionnaires were completed. We excluded 10 participants: three did not complete the questionnaire, three wrote their names instead of their age, two had missing data for age, one wrote her age as '240' and one participant chose the first option for each question. This left 403 valid questionnaires for analysis, giving an effective recovery rate of 96.4%. Detailed data collection procedures are presented in supplementary figure 1.

Data processing and analysis

We used SPSS version 25.0 (IBM Corp., Armonk, NY) for all data analyses. Descriptive statistics were calculated for baseline characteristics and categorical variables using simple frequencies and percentages. The main outcome variables were knowledge, attitudes and practices. The knowledge score for each participant was determined by allotting a score of '1' to correct responses and '0' (zero) to incorrect and 'do not know' responses. Therefore, the maximum obtainable knowledge score was 16. A knowledge score ≤10 was considered poor knowledge, and scores >10 represented good knowledge.[22] The attitude scores were obtained by assigning points to responses on the 5-point Likert scale (1 point for 'strongly disagree' to 5 points for 'strongly agree'). The maximum obtainable attitude score was 35 points. An attitude score ≤28 was considered a poor attitude and scores >29 were categorised as good attitudes.[22] Similarly, the practice score for each participant was determined by allotting a score of '1' to correct responses and '0' (zero) to incorrect responses. This gave a maximum obtainable practice score of 4. Those with a practice score <4 were regarded as having poor practice, whereas a score of 4 was considered to reflect good practice.[22] Finally, the levels of knowledge, attitude and practice were coded as 0 for poor, 1 for good.[22] Chi-square tests (bivariable analyses) were used to determine the associations between the baseline and outcome variables. All variables with $P<0.05$ in the bivariable analysis were included in the binary logistic regression (multivariable analysis) to determine the associations between dependent (knowledge, attitudes and practices) and

independent (baseline characteristics) variables. In consideration of having yuesao or not is related to salary range and education, which are supposed to affect attitute and knowledge towarding to neonatal jaundice.Thus, another comparison had been done to differentiate those without yuesao and with yuesao in correlation to education, salary in terms of attitute and knowledge. Statistical significance was represented by $P<0.05$ at a 95% confidence level.

Patient and public involvement

Before the formal survey, the researchers interviewed 20 mothers to determine the readability of the questionnaire, the time required and the best way to conduct the investigation.

RESULTS

Participants' baseline characteristics

Of the 403 mothers included in our analyses, a majority were aged 28~32 years (48.9%) and 75.7% had a spontaneous vaginal delivery. Most mothers (80.6%) had received prior health education about jaundice from health workers on the day of normal discharge from the delivery hospital (48 to 72 hours after vaginal delivery or 96 to 120 hours after caesarean delivery). Among the 403 participating mothers, 113 (28%) reported their current child was admitted to the hospital for treatment due to jaundice after discharge, and 56 (13.9%) had a previous child with a history of neonatal jaundice. Participants' baseline characteristics are presented in Table 1.

Table 1. Baseline characteristics of participating mothers (N=403)

Variables	Characteristics	n (%)
**Sociodemographic data**		
	19~27	114 (28.3)
Age, years	28~32	197 (48.9)
	33~45	92 (22.8)
	O	145 (36.0)
Blood group	A	121 (30.0)
	B	106 (26.3)
	AB	31 (7.7)
	High school and below	68 (16.9)
Education level	University	310 (76.9)
	Postgraduate and above	25 (6.2)
	Employed	267 (66.3)
Occupation	Self-employed	40 (9.9)
	Homemaker	82 (20.3)

For peer review only

	Others	14 (3.5)
Average family monthly income, RMB	≤5000	50 (12.4)
	5001~10000	154 (38.2)
	10001~20000	125 (31)
	20001~30000	35 (8.7)
	≥30001	39 (9.7)
Time from the place of residence to the delivery hospital, minutes	≤10	52 (12.9)
	10~30	212 (52.6)
	30~60	126 (31.3)
	≥60	13 (3.2)
**Delivery history**		
Parity	Primipara	224 (55.6)
	Multipara	179 (44.4)
Delivery mode	Spontaneous vaginal	305 (75.7)
	Caesarean section	98 (24.3)
**Infant's Information**		
Sex	Male	210 (52.1)
	Female	193 (47.9)
Birth weight[1]	Low	365 (7.7)
	Normal	31 (90.6)
	Hight	7 (1.7)
Feeding method	Exclusive breastfeeding	196 (48.6)
	Mixed feeding	197 (48.9)
	Exclusive formula-feeding	10 (2.5)
Cranial haematoma[2]	Yes	15 (3.7)
	No	364 (90.3)
	Not sure	24 (6.0)
Whether meconium passed within 24 hours	Yes	397 (98.5)
	No	6 (1.5)
Predischarge bilirubin level	Normal	312 (77.4)
	Height	91 (22.6)
'Yuesao'[3]	Yes	138 (34.2)
	No	265 (65.8)
**Previous experience/exposure to neonatal jaundice**		
Prior health education on neonatal jaundice	Yes	361 (80.6)
	No	42 (10.4)
Family history/friends with neonatal jaundice history(N=373)	Yes	45 (12.1)
	No	328 (87.9)
Previous child with a history of neonatal jaundice	Yes	56 (13.9)
	No	347 (86.1)

For peer review only

Current child admitted to hospital for treatment for jaundice after discharge	Yes	113 (28.0)
	No	290 (72.0)
Mother's knowledge level	Good	183 (45.4)
	Poor	220 (54.6)
Mother's attitude level	Good	170 (42.2)
	Poor	233 (57.8)
Mother's practice level	Good	214 (53.1)
	Poor	183 (46.9)

Note: [1] Weight: low weight <2500 g; normal weight 2500–4000 g; high weight >4000 g. [2] Cranial haematoma: haematoma caused by rupture and bleeding of subperiosteal vessels in the parieto-occipital region due to birth injury. [3] *yuesao*: maternity matron specialised in caring for mothers and newborns.

Mothers' knowledge of neonatal jaundice

We found that 45.4% of participating mothers had good knowledge regarding neonatal jaundice. The rates of correct answers to the knowledge questions ranged from 29% to 96.8%. Questions that showed low rates of correct answers were: 'Cranial haematoma may cause neonatal jaundice' (29%), 'Blood test is the gold standard for diagnosing neonatal jaundice' (34.2%), 'It is abnormal for jaundice to appear within 24 hours after birth' (36.2%), 'It is abnormal for jaundice to reappear after it subsides' (37%), and 'Breast milk jaundice is a benign and self-limited condition, and interruption of breast feeding is not recommended as a therapeutic intervention' (37%). Table 2 presents scores for knowledge about neonatal jaundice among participating mothers.

Table 2. Maternal knowledge about neonatal jaundice (N=403)

**Items**	**True n (%)**	**False n (%)**	**Don't know n (%)**	**Correct rate n (%)**
**Observation of neonatal jaundice**				
When newborns develop jaundice, their skin will turn yellow.	390 (96.8)	4 (1.0)	9 (2.2)	390 (96.8)
When newborns develop jaundice, their face will turn yellow first.	344 (85.4)	12 (3.0)	47 (11.7)	344 (85.4)
When looking for jaundice, check the naked baby in bright and preferably natural light.	365 (90.6)	10 (2.5)	28 (6.9)	365 (90.6)
**Classification of neonatal jaundice**				
Neonatal jaundice is divided into physiological	371 (92.1)	3 (0.7)	29 (7.2)	371 (92.1)

For peer review only - http://bmjopen.bmj.com/site/about/guidelines.xhtml

jaundice and pathological jaundice.				
**Danger signs of neonatal jaundice**				
Palms and soles turn yellow, indicating that jaundice is severe.	265 (65.8)	29 (7.2)	109 (27.0)	265 (65.8)
It is an abnormal condition if the jaundice appears within first 24 h.	146 (36.2)	155 (38.5)	102 (25.3)	146 (36.2)
It is an abnormal condition that the jaundice reappears after it has subsided.	149 (37.0)	143 (35.5)	111 (27.5)	149 (37.0)
**Complication of neonatal jaundice**				
Severe jaundice may lead to brain damage.	325 (80.6)	7 (1.7)	71 (17.6)	325 (80.6)
**Cause of neonatal jaundice**				
The mother's blood type is O, and the father's blood type is A, B, or AB, which may cause neonatal jaundice.	228 (56.6)	38 (9.4)	137 (34.0)	228 (56.6)
Cranial hematoma may cause neonatal jaundice.	117 (29.0)	39 (9.7)	247 (61.3)	117 (29.0)
Bowel obstruction may cause neonatal jaundice.	281 (69.7)	14 (3.5)	108 (26.8)	281 (69.7)
Broad bean disease (G6PD) may cause jaundice.	163 (40.4)	25 (6.2)	215 (53.3)	163 (40.4)
Breastfeeding may cause jaundice	200 (49.6)	109 (27.0)	94 (23.3)	200 (49.6)
**Breast milk jaundice**				
Breast milk jaundice is a benign and self-limited condition, and interruption of breast feeding is not recommended as a therapeutic intervention.	149 (37.0)	135 (33.5)	119 (29.5)	149 (37.0)
**Diagnosis of neonatal jaundice**				
Blood test is the gold standard for diagnosing neonatal jaundice.	138 (34.2)	127 (31.5)	138 (34.2)	138 (34.2)
**Treatment of neonatal jaundice**				
Phototherapy is a common, effective and safe treatment method for neonatal jaundice.	367 (91.1)	3 (0.7)	33 (8.2)	367 (91.1)

Mothers' attitudes towards neonatal jaundice

The results revealed that 42.2% of participating mothers had poor attitudes towards neonatal jaundice. Over half of the participants strongly agreed that post-discharge observation was necessary and post-partum visits were needed to measure the bilirubin level (52.4% and 51.9%, respectively). However, some mothers believed that neonatal

For peer review only

jaundice was a common physiological phenomenon that would not cause serious consequences (10.9%), and 29.2% lacked understanding that adequate feeding was conducive to resolving jaundice. Mothers' attitudes towards neonatal jaundice are presented in Table 3.

Table 3. Maternal attitudes towards neonatal jaundice (N=403)

Items	Strongly disagree n (%)	Disagree n (%)	Not sure n (%)	Agree n (%)	Strongly agree n (%)
I think neonatal jaundice is a common physiological phenomenon and will not cause serious consequences. (-)	80 (19.1)	216 (53.6)	63 (15.6)	39 (9.7)	5 (1.2)
I think that a baby with jaundice, does not need treatment and will self-recover. (-)	151 (38.5)	201 (49.9)	43 (10.7)	6 (1.5)	2 (0.5)
I think it is very important to observe neonatal jaundice after discharged from the hospital.	5 (1.2)	0 (0.0)	6 (1.5)	181 (44.9)	211 (52.4)
I think it is necessary for post-partum visitors to assess jaundice condition.	8 (1.0)	0 (0.0)	8 (2.0)	182 (45.2)	209 (51.9)
I think a baby with suspected jaundice should go to a medical institution or community healthcare centre to measure the bilirubin level in a timely manner.	3 (0.7)	5 (1.2)	10 (2.5)	230 (57.1)	155 (37.5)
I believe that adequate breastfeeding is good for jaundice.	5 (1.2)	29 (7.2)	84 (20.8)	196 (48.6)	89 (22.1)
I think it is necessary to learn knowledge of neonatal jaundice.	2 (0.5)	0 (0.0)	10 (2.5)	216 (53.6)	175 (43.4)

Note: - represents reverse scored items.

Mothers' practices regarding neonatal jaundice

In general, 53.1% of mothers had good practices regarding neonatal jaundice, and 96% checked their baby for jaundice after discharge. However, only 41.9% indicated they would take the initiative to learn about neonatal jaundice after discharge. Mothers' practices regarding jaundice are shown in Table 4.

Table 4. Maternal practices regarding neonatal jaundice (N=403)

For peer review only

Items	Yes<br>n (%)	No<br>n (%)
I took the initiative to seek information about neonatal jaundice.	168 (41.7)	235 (58.3)
After discharge, I checked my infant for jaundice, such as the colour of the skin, sclerae, urine, bowel movements.	387 (96.0)	16 (4.0)
After discharge, I followed the doctor's instructions to take the infant to a medical institution or community healthcare centre to measure the bilirubin level.	376 (93.3)	27 (6.7)
After discharge, I breastfed adequately.	359 (89.1)	44 (10.9)

Factors associated with knowledge, attitudes and practices related to neonatal jaundice among mothers

The binary logistic regression analysis revealed that good knowledge about jaundice was associated with a high level of education (master's degree or above; odds ratio [*OR*]=5.977, 95% confidence interval [*CI*]: 1 .994~17.916, *P*=0.001), receiving prior health education on neonatal jaundice (*OR*=3.617, 95%*CI*: 1.637~7. 990, *P*=0.001), and male babies (*OR*=1.714, 95%*CI*: 1.122~2.167, *P*=0.013). A positive attitude towards ja undice was associated with being cared for by a '*yuesao*' (matron specialised in maternal and newborn ca re) (*OR*=1.969, 95%*CI*: 1.264~3.066, *P*=0.003) and good knowledge about jaundice (*OR*=1.804, 95%*CI*: 1. 194~2.726, *P*=0.005). Finally, good practices related to jaundice were associated with prior health educatio n on neonatal jaundice (*OR*=2.260, 95%*CI*: 1.105~4.625, *P*=0.026) and good knowledge about jaundice (*O R*=3.112, 95%*CI*: 2.040~4.749, *P*<0.001). The results of chi-square tests (bivariable analyses) for maternal knowledge, attitudes and practices related to neonatal jaundice are shown in supplementary table 1. Factor s associated with knowledge, attitudes and practices related to neonatal jaundice among mothers are shown in Table 5. Binary logistic regression analysis of maternal knowledge, attitudes and practices related to ne onatal jaundice for mothers with and without yuesao are shown in supplementary table 2 and supplementa ry table 3.

For peer review only

Table 5. Binary logistic regression analysis of maternal knowledge, attitudes and practices related to neonatal jaundice (N=403)

Variables	Classification	Knowledge			Attitude			Practices		
		OR	*95%CI*	*P*	*OR*	*95%CI*	*P*	*OR*	*95%CI*	*P*
Education level	High school and below (Ref.)			**0.001**		*NI*			*NI*	
	College and undergraduate course	3.011	1.563~5.800	0.001						
	Postgraduate student or above	5.977	1.994~17.916	0.001						
Average family monthly income (RMB)	≤5000 (Ref.)		*NI*			*NI*			*NI*	
	5001~10000									
	10001~20000									
	20001~30000									
	≥30001									
Occupation	Employed (Ref.)			0.110		*NI*			*NI*	
	Self-employed	0.403	0.183~0.887	0.024						
	Homemaker	0.730	0.409~1.302	0.286						
	Other	1.266	0.401~3.994	0.688						
Parts	Primipara (Ref.)		*NI*						*NI*	
	Multipara				1.498	0.995~2.255	0.053			
Prior education on neonatal jaundice	No (Ref.)					*NI*				
	Yes	3.617	1.637~7.993	**0.001**				2.260	1.105~4.625	**0.026**
Predischarge bilirubin	Normal		*NI*			*NI*			*NI*	

For peer review only

level	Hight									
Neonate sex	Female (Ref.)					*NI*			*NI*	
	Male	1.714	1.122~2.617	**0.013**						
Cranial hematoma	No (Ref.)			0.073						
	Yes	1.549	0.510~4.706	0.440		*NI*			*NI*	
	It is not clear	0.321	0.112~0.920	0.034						
Yuesao	No (Ref.)		*NI*						*NI*	
	yes				1.969	1.264~3.066	**0.003**			
Knowledge level	Poor (Ref.)		*NA*							
	good				1.804	1.194~2.726	**0.005**	3.112	2.040~4.749	**0.000**
Attitude level	poor (Ref.)									
	good		*NI*			*NA*		1.498	0.983~2.283	0.060

Note: *OR,* odds ratio; *CI* confidence interval; *NI*, not included in the final logistic regression analysis; *NA*, not applicable.

For peer review only

DISCUSSION

In this study, 45.4% of mothers had good knowledge about neonatal jaundice. This knowledge level was better than that reported in other countries such as Nepal[23] (22%), Egypt [24] (30%) and Karbala city, Iraq[25] (34%). This may be attributed to the high proportion (83.1%) of our respondents who had graduated from university. Our study also found that education level was significantly associated with knowledge about neonatal jaundice, which was consistent with the results of an earlier review.[26] Due to differences in scoring algorithms or items investigating attitudes and practices[23–25], it was difficult to compare attitudes and behaviours reported in our study with those from other countries.

The majority (80.6%) of participants in this study had received prior health education on neonatal jaundice, which suggested that their knowledge about neonatal jaundice would be high. However, this study found that many mothers had poor knowledge regarding neonatal jaundice, with only 45.5% of participating mothers had good knowledge about neonatal jaundice. This large gap may be attributable to the gap of nearly a week between the time they received health education and the time of our investigation; some mothers might have forgotten the content of the health education. In addition, medical staff only provided post-discharge monitoring and follow-up instruction and did not include neonatal jaundice knowledge related to the questionnaire when conducting health education. Another factor that might have contributed to the comparative ineffectiveness of postnatal instruction was that the unique environment was absent that mothers received health education about jaundice from health workers in a single setting, which combined a lecture, demonstration and interactive discussion, as there is generally a rush to discharge mothers from birthing centres.

The present study reported that many mothers had poor knowledge regarding the causes and danger signs of jaundice. Relatively few mothers provided correct answers to some items; for example, ‘Cranial haematoma may cause neonatal jaundice’ (29%), ‘Blood test is the gold standard for diagnosing neonatal jaundice’ (34.2%), ‘It is abnormal for jaundice to appear within 24 hours after birth’ (36.2%), and ‘It is abnormal for jaundice to reappear after it subsides’ (37%). Poor knowledge about the causes and danger signs of jaundice may mean mothers turn to traditional treatments, which results in delays in seeking medical attention for neonatal jaundice, thereby contributing to the development of ABE and kernicterus.[11] Therefore, it is recommended that medical staff incorporate information about the causes

For peer review only

and danger signs of jaundice into neonatal jaundice health education programmes. For breast milk jaundice, this involves monitoring the jaundice without changing in the infant's breastfeeding;[27] however, 73% of mothers in this study did not know that breast milk jaundice is generally a benign condition, where interruption of breast feeding is not recommended as a therapeutic intervention. Having poor knowledge of breast milk jaundice may mean mothers discontinue breastfeeding after jaundice occurs. However brief, such discontinuation may jeopardise an infant's ability to return to exclusive breastfeeding, which is unnecessarily harmful to the infant and traumatic for mothers.[28] This means mothers of affected infants should be educated about breast milk jaundice and informed that breastfeeding should be interrupted in rare instances (i.e. if the neonate displays signs of ABE). In addition, the incidence of G6PD deficiency is high in Shenzhen.[29–30] Traditional Chinese medicine is widely used in China to prevent or treat neonatal jaundice.[31–32] However, neonates with G6PD deficiency that use such remedies may have severe jaundice.[33] Our findings suggested that most mothers (59.5%) did not know the G6PD deficiency may cause jaundice. Therefore, mothers whose neonates have G6PD deficiency should be educated about avoiding using traditional Chinese medicine to treat or prevent neonatal jaundice.

Importantly, our study highlighted that maternal information seeking behaviour related to neonatal jaundice needs to be improved. Only 41.7% of mothers in this study indicated they took the initiative to seek information about neonatal jaundice. This poor practice could be because physical and psychological changes in the postpartum period mean mothers' energy is limited. However, it could also be attributable to health literacy, which has an impact on people's health information seeking behaviour.[34] A previous study[34] reported that the lower the parents' health literacy, the less likely they were to take the initiative to obtain information about their child's health. Actively understanding relevant knowledge will help to improve maternal awareness of neonatal jaundice, which will be conducive to managing neonatal jaundice after discharge from hospital. In addition, with the popularisation of the Internet and smart phones, more parents are using these ways to access parenting knowledge. However, they complain that they face major challenges in identifying whether the information is reliable.[35-36] Therefore, it is recommended that when providing education about neonatal jaundice, medical staff also provide mothers with some reliable websites to facilitate the active information seeking about neonatal jaundice.

Our multivariate analysis revealed that mothers who had a male infant were more likely be

For peer review only

knowledgeable about neonatal jaundice compared with mothers who had a female infant. Health workers generally perform routine jaundice evaluation during birth hospitalisation, and male sex is a risk factor for neonatal jaundice.[37] We speculated that mothers who gave birth to male infants had more opportunities to receive information about neonatal jaundice. A high education level was determinant of knowledge about neonatal jaundice. This finding was consistent with the results of a study from Egypt[13] that found mothers who were university graduates had the highest knowledge scores. This suggested it is necessary for medical staff to provide information about jaundice to mothers with lower education levels. Interestingly, for the mothers without yuesao, the binary logistic regression analysis revealed that good knowledge about jaundice was associated with receiving prior health education on neonatal jaundice from medical staff. However, for the mothers with yuesao, no statistically significant association was detected between prior health education on neonatal jaundice and knowledge towards jaundice. This may be because the yuesao offers a valuable resource for jaundice counselling. Chinese tradition dictates that new mothers stay home and rest for a 'confinement period' of about 1 month (28 to 42 days) after giving birth, which is thought to facilitate recovery. Previous studies reported that being cared for by a yuesao during this stage can help improve the health of mothers and babies,[38-39] reduce postpartum depression,[40-41] and facilitate breastfeeding.[42] Therefore, hiring a yuesao to prepare post-partum foods and help with household and childcare tasks has become increasingly popular in many urban families. However, not everyone can afford their services. This suggested it is necessary for hospitals to give mothers pamphlet on neonatal jaundice in which informations on recommended websites to seek further information can be obtained.This will help mothers especially those who cannot afford a yuesao.

Our multivariate analysis of practices related to neonatal jaundice revealed that mothers who had received prior education on neonatal jaundice from medical staffs were more likely to have good practices related to jaundice than other mothers. This finding was consistent with a study conducted in Nigeria[43] that showed mothers who obtained knowledge about neonatal jaundice from medical staff were significantly less likely to self-treat and more likely to seek medical treatment promptly. We also found that mothers with good knowledge about neonatal jaundice were more likely to have good attitudes and practices; this was consistent with the 'knowledge, attitudes practices' (KAP) model,[44] which suggests greater knowledge is

For peer review only

the basis for good attitudes and practices.

LIMITATIONS

This study had some limitations. One limitation was that when recruiting mothers, we might have motivated them to learn about neonatal jaundice, which would have improved mothers' awareness of neonatal jaundice before the investigation. However, Chinese traditional culture indicates that mothers need to confine themselves for 1 month after giving birth, so they do not leave their homes during this period. This also made it difficult to recruit mothers after discharge from the hospital. To increase the accessibility of the population, this study recruited mothers in advance during the hospitalisation period after delivery. Another limitation was that our research findings are only representative of mothers' KAP, but for some infants, the main caregivers are other people such as grandmothers, sisters or aunts. The findings of this study are not representative of these individuals.

CONCLUSION

Overall, mothers' knowledge about jaundice was low, especially regarding causes, danger signs and breast milk jaundice. Active information seeking behaviour about neonatal jaundice needs to be improved. Therefore, it is recommended that medical staff incorporate information about the causes, danger signs and breast milk jaundice into neonatal jaundice health education programmes and provide reliable websites for mothers to obtain information about neonatal jaundice. This study also showed that the mother's education level was an important factor that is significantly associated with knowledge about jaundice. In addition, mothers receiving care from a *yuesao* tend to have positive attitudes toward jaundice. Enhancing jaundice-related education programmes targeting mothers with a low education level and no *yuesao* care is important.

Acknowledgments: We would like to acknowledge seven neonatologists and my research team for giving feedback to the questionnaire development. Our appreciation also goes to the study participants for generously spending time and providing information in this survey.

Contributors: Y.H. and L.C. are joint first authors. Y.H., L.C., and X.J.W. designed the study. Y.H., X.J.W., C.Z., Z.L.G., F.Y., and J.L. collected the data. Y.H., L.C., and X.J.W. analyzed the data. Y.H drafted the manuscript. W.Z.C., Y.H. and L.C. contributed to the interpretation of the results and critical revision of the manuscript for important intellectual content and approved the final version of the

For peer review only

manuscript. All authors have read and approved the final manuscript. W.Z.C.,Y.H.,and L.C.are the study guarantors.

Funding: This study was supported by grants from Sanming Project of Medicine in ShenZhen, China (SZSM201612018).

Competing interests: None declared.

Patient consent for publication: Not aplicable.

Data availability statement: All data relevant to the study are included in the article or uploaded as supplementary information.

Ethics approval: Ethical approval was obtained from the institutional ethics board of Shenzhen Hospital of Southern Medical University (approval number: NYSZYYEC20210004).

REFERENCES

1. Wang WP. Pediatrics. Poeple's Medical Publishing House. 2018.

2. Management of hyperbilirubinemia in the newborn infant 35 or more weeks of gestation. Pediatric s 2004;114(1):297-316.

3. Riordan SM, Shapiro SM. Review of bilirubin neurotoxicity I: molecular biology and neuropatholo gy of disease. Pediatr Res 2020;87(2):327-31.

4. Karimzadeh P, Fallahi M, Kazemian M, et al. Bilirubin Induced Encephalopathy. Iran J Child Neu rol. 2020;14(1):7-19.

5. Ma XL, Shi LP, Du LZ, et al. Clinical characteristics of bilirubin encephalopathy in Chinese newb orn infants-a national multicenter survey. Zhonghua Er Ke Za Zhi 2012;50(05): 331-335.

6. Bhutani VK, Zipursky A, Blencowe H, et al. Neonatal hyperbilirubinemia and Rhesus disease of t he newborn:incidence and impairment estimates for 2010 at regional and global levels. PEDIATRRE S 2013;74:86-100.

7. Wennberg RP, Oguche S, Imam Z, et al. Maternal Instruction About Jaundice and the Incidenceof Acute Bilirubin Encephalopathy in Nigeria. J Pediatr 2020;221:47-54.

8. Dlala UM, Wennberg RP, Abdulkair I, et al. Patterns of acute bilirubin encephalopathy in Nigeria: a multicenter pre-intervention study. J Perinatol 2018;38(7):873-880.

For peer review only

9. Addendum to Jaundice in newborn babies under 28 days. London: National Institute for Health and Care Excellence (UK). 2016.

10. Watchko JF. Maternal Instruction on Neonatal Jaundice: What Can we Learn from the Stop Kernicterus in Nigeria (SKIN) Experience? J Pediatr 2020;221:7-8.

11. Salia SM, Afaya A, Wuni A, et al. Knowledge, attitudes and practices regarding neonatal jaundice among caregivers in a tertiary health facility in Ghana. PloS one 2021;16(6).

12. Amegan-aho KH, Segbefia CI, Glover N, et al. Neonatal Jaundice: awareness, perception and preventive practices in expectant mothers. Ghana Med J 2019;53(4):267-272.

13. Moawad EMI, Abdallah EAA, Ali YZA. Perceptions, practices, and traditional beliefs related to neonatal jaundice among Egyptian mothers. Medicine 2016;95(36):e4804.

14. Editorial Board of Chinese Journal of Pediatrics Subspecialty Group of Neonatology, The Society of Pediatrics, Chinese Medical Association. [Experts consensus on principles for diagnosis and treatment of neonatal jaundice]. Zhonghua Er Ke Za Zhi 2010;48:685–686.

15. Adoba P, Ephraim RKD, Kontor KA, et al. Knowledge Level and Determinants of Neonatal Jaundice: A Cross-Sectional Study in the Effutu Municipality of Ghana. Int J Pediatr 2018.

16. Demis A, Getie A, Wondmieneh A ,et al. Knowledge on neonatal jaundice and its associated factors among mothers in northern Ethiopia: a facility-based cross-sectional study. BMJ Open 2021;11(3):e44390.

17. Shi JC, Mo XK, Sun ZQ. Content validity index in scale development. Zhong Nan Da Xue Xue Bao Yi Xue Ban 2012;37(02):49-52.

18. Mcneish D. Thanks coefficient alpha, we'll take it from here. Psychological Methods 2018;23(3):412-433.

19. Li Z. Nursing Research Methods[M]. Poeple's Medical Publishing House. 2018.

20. Cronbach LJ. Coefficient alpha and the internal structure of tests. Psychometrika 1951;16(3):297-334.

21. Clark LAWD. Constructing validity: Basic issues in objective scale development. Psychol Assess 1995;7(3):309-19.

For peer review only

22. Li TT, Wang J, Chen X, Chen L, et al.Obstetric Nurses' Knowledge, Attitudes, and Professional Support Related to Actual Care Practices About Urinary Incontinence. Female Pelvic Med Reconstr S urg 2021;27(2):e377-384.

23. Shrestha S, Maharjan S, Petrini M. Knowledge about neonatal jaundice among Nepalese mothers. JBPKIHS 2019;2:34-42.

24. Kasemy ZA, Bahbah WA, El Hefnawy SM, et al. Prevalence of and mothers' knowledge, attitud e and practice towards glucose-6-phosphate dehydrogenase deficiency among neonates with jaundice: a cross-sectional study. BMJ Open 2020;10(2): e34079.

25. Hussein H, Aziz A. Assessment of mothers' knowledge and beliefs toward care of neonatal jaun dice in pediatric teaching hospital in Holy Karbala City. Int J Sci Res 2016;6.

26. Wennberg RP, Watchko JF, Shapiro SM. Maternal Empowerment - An Underutilized Strategy to Prevent Kernicterus? Curr Pediatr Rev 2017;13(3):210-219.

27. Bratton S, Cantu RM, Stern M, et al. Breast Milk Jaundice(Nursing). 2022.

28. Preer GL, Philipp BL. Understanding and managing breast milk jaundice. Arch Dis Child Fetal Neonatal Ed 2011;96(6):F461-6.

29. Gao J, Lin S, Chen S, et al. Molecular Characterization of Glucose-6-Phosphate Dehydrogenase Deficiency in the Shenzhen Population. Hum Hered 2020;85(3-6):110-116.

30. Liu Z, Yu C, Li Q, et al. Chinese newborn screening for the incidence of G6PD deficiency and variant of G6PD gene from 2013 to 2017. Hum Mutat 2020;41(1):212-221.

31. Standardization Project Team of Guidelines for Clinical Application of Chinese Patent Medicines for the Treatment of Predominant Diseases. Guidelines for clinical application of Chinese patentmedic ine in the treatment of neonatal jaundice (2020). Chin J Integr Tradit Chin West Med 2021;41(03):28 0-285.

32. Gu Minyong, Yang Yan, Sun Yanli, et al. Guidelines for Clinical Diagnosis and Treatment ofPe diatrics in Traditional Chinese Medicine · Neonatal jaundice (Revised). Pediatrics Journal of Tradition al Chinese Medicine 2018;14(02):5-9.

33. Fok TF. Neonatal jaundice--traditional Chinese medicine approach. *J Perinatol*, 2001;21 Suppl 1: S98-S100, S104-S107.

For peer review only

34. Sørensen K, Van den Broucke S, Fullam J, et al. Health literacy and public health: a systematic review and integration of definitions and models. BMC Public Health 2012;12:80.

35. Kubb C, Foran HM. Online Health Information Seeking by Parents for Their Children: Systemati c Review and Agenda for Further Research. J Med Internet Res 2020;22(8):e19985.

36. Smith CA, Parton C, King M, et al. Parents' experiences of information-seeking and decision-ma king regarding complementary medicine for children with autism spectrum disorder: a qualitative stud y. BMC Complement Med Ther 2020;20(1):4.

37. Birhanu MY, Workineh AA, Molla Y, et al. Rate and Predictors of Neonatal Jaundice in Northw est Ethiopia: Prospective Cohort Study. J Multidiscip Healthc 2021;14:447-457.

38. Li YY, Wang HY. The impact of yuesao on the health of mothers and infants. Chinese JounalOf Practical Actical Nursing 2010;26(27): 42-43.

39. Yang L, Zhu MY, Yin Y. Impact of babysitter's care on health status of infants: a survey study. Journal of Nursing Science 2011;(18):87-88.

40. Xing CG. Practical Strategies and Policy Implications of Postpartum Doulas' Intervention on Post partum Depression. Medicine and Society 2021;34(04):11-16.

41. Peng K, Zhou L, Liu X, et al. Who is the main caregiver of the mother during the doing-the-mo nth: is there an association with postpartum depression? BMC Psychiatry 2021;21(1):270.

42. Wu W, Zhang J, Silva ZI, et al. Factors influencing breastfeeding practices in China: A meta-agg regationof qualitative studies. Matern Child Nutr 2021:e13251.

43. Ezeaka CV, Ugwu RO, Mukhtar-Yola M, et al. Pattern and predictors of maternal care-seekingpr actices for severe neonatal jaundice in Nigeria: a multi-centre survey. BMC Health Serv Res 2014;14: 192.

44. Badran IG. Knowledge, attitude and practice the three pillars of excellence and wisdom: a place in themedical profession. 1995.

Figure legend

Supplementary figure 1 data collection procedures

For peer review only

For peer review only

For peer review only

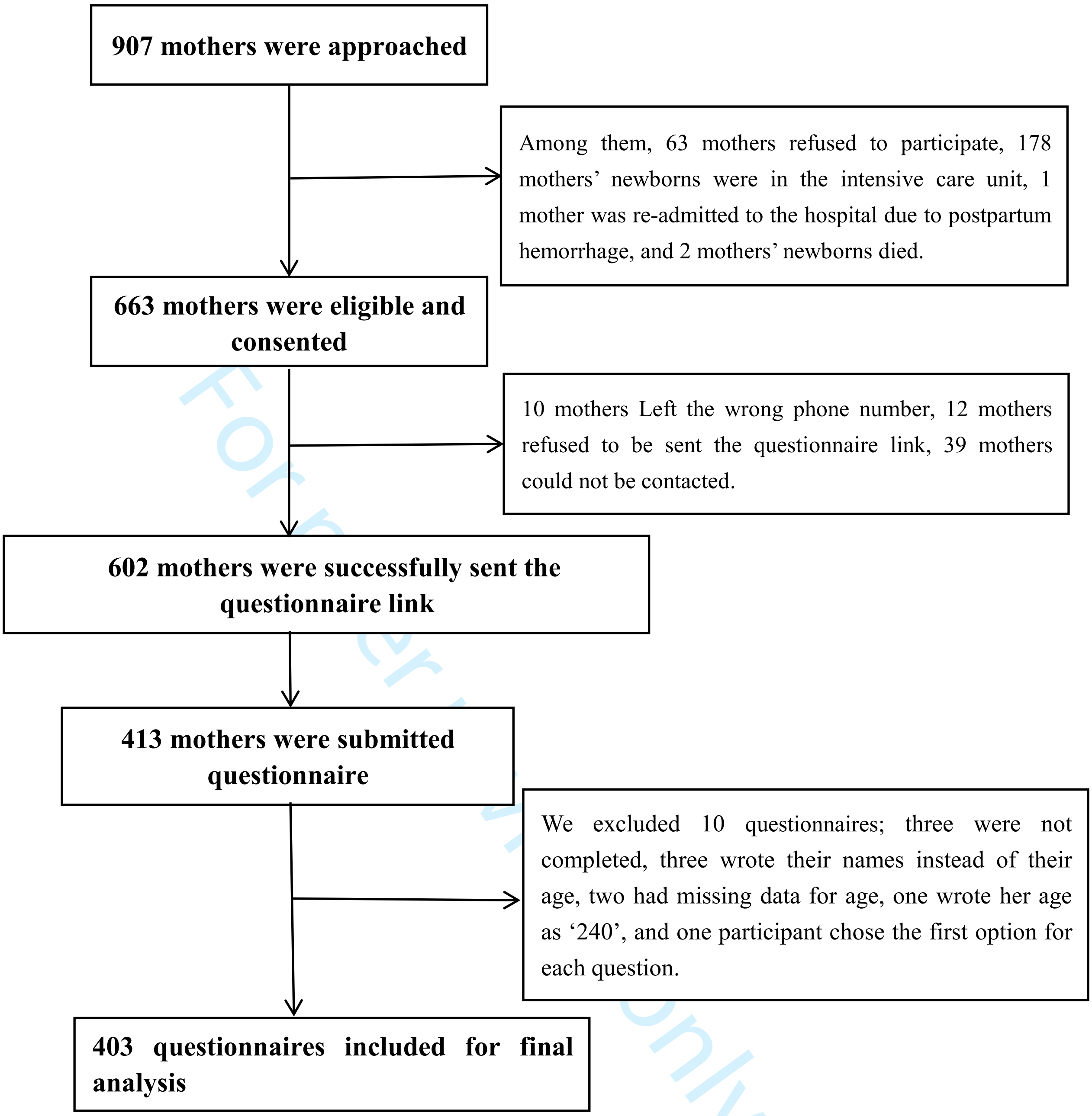

For peer review only

Supplementary table 1. Chi-square analyses of maternal knowledge, attitudes and practices related to neonatal jaundice (N=403)

Variables		Knowledge*		Attitude*		Behaviour*	
		χ^2	*p*	χ^2	*p*	χ^2	*p*
**Sociodemographic data**							
Age(years)	19~27<br>28~32<br>33~45	2.008	0.366	1.385	0.500	0.292	0.864
Blood group	O<br>A<br>B<br>AB	1.668	0.644	3.873	0.276	4.244	0.236
Education level	High school and below<br>University<br>Postgraduate and above	**16.921**	**0.000**	**9.800**	**0.007**	**6.084**	**0.048**
Occupation	Employed<br>Self-employed<br>Housewife<br>Others	**15.279**	**0.002**	2.007	0.571	6.865	0.076
Average family monthly income (RMB)	≤5000<br>5001~10000<br>10001~20000<br>20001~30000<br>≥30001	**10.444**	**0.034**	6.849	0.144	8.683	0.070
Time from the place of residence to the delivery hospital (minutes)	≤10<br>10~30<br>30~60<br>≥60	1.458	0.692	3.289	0.349	1.635	0.652
**Delivery history**							
Parity	Primiparous<br>Multipara	0.021	0.885	**4.536**	**0.033**	0.629	0.428
Delivery mode	Spontaneous vaginal<br>Caesarean section	0.014	0.907	0.152	0.696	0.500	0.479
**Infant's Information**							
Sex	Male<br>Female	**6.409**	**0.011**	2.241	0.134	0.804	0.370

For peer review only

Birth weight[1]	Low<br>Normal<br>Hight	1.008	0.615	2.034	0.402	4.216	0.130
Feeding way	Exclusive breastfeeding<br>Mixed feeding<br>Exclusive Formula-feeding	5.479	0.065	0.873	0.647	5.604	0.062
Cranial hematoma[2]	Yes<br>No<br>Not sure	**7.288**	**0.026**	0.342	0.843	0.380	0.827
Whether to pass meconium with in 24 hours	Yes<br>No	0.052	0.820	1.626	0.202	3.247	0.072
Predischarge bilirubin level	Normal<br>Hight	1.253	0.263	0.022	0.882	1.837	0.175
"yuesao" [3]	Yes<br>No	1.067	0.320	**11.878**	**0.001**	0.327	0.567
**Previous experience/exposure to NNJ**							
Prior health education on neonatal jaundice	Yes<br>No	**8.824**	**0.003**	0.179	0.672	**9.236**	**0.002**
Previous child history of neonataljaundice	Yes<br>No	1.067	0.302	0.140	0.708	0.133	0.715
Current child admitted to the hospital for treatment due to jaundice after disch arge	Yes<br>No	0.358	0.549	0.012	0.912	3.156	0.076
Family history/friends with NNJ history（N=373)	Yes<br>No	0.351	0.554	0.100	0.752	0.860	0.354
Mother's knowledge level	good<br>poor	*NA*		**9.478**	**0.002**	**35.751**	**0.000**
Mother's attitude level	good<br>poor	*NA*		*NA*		**7.179**	**0.007**

Note: *NA*, not applicable. *Knowledge attitude and behaviour were all divided into two levels (po or or good)

For peer review only

Supplementary table 2. Binary logistic regression analysis of maternal knowledge, attitudes and practices related to neonatal jaundice for mothers with yuesao (N=138)

Variables	Classification	Knowledge			Attitude			Practices		
		OR	*95%CI*	*P*	*OR*	*95%CI*	*P*	*OR*	*95%CI*	*P*
Education level	High school and below (Ref.)			**0.048**			**0.033**		*NI*	
	College and undergraduate course	7.123	1.387~36.588	0.019	7.683	1.583~37.297	0.011			
	Postgraduate student or above	4.316	0.645~28.877	0.132	9.900	1.539~63.689	0.016			
Average family monthly income (RMB)	≤5000 (Ref.)		*NI*			*NI*			*NI*	
	5001~10000									
	10001~20000									
	20001~30000									
	≥30001									
Prior education on neonatal jaundice	Yes (Ref.)		*NI*			*NI*			*NI*	
	No									
Neonate sex	Female (Ref.)		*NI*			*NI*			*NI*	
	Male									
Occupation	Employed (Ref.)			**0.017**					*NI*	
	Self-employed	0.131	0.034~0.496	0.003		*NI*				

For peer review only

Variables	Classification	Knowledge OR	Knowledge 95%CI	Knowledge P	Attitude OR	Attitude 95%CI	Attitude P	Practices OR	Practices 95%CI	Practices P
	Homemaker	1.182	0.393~3.559	0.766						
	Other	0.306	0.390~0.064	0.306						
Knowledge level	Poor (Ref.)	*NA*			*NI*					
	good							0.505	0.254~1.003	0.051
Attitude level	poor (Ref.)	*NI*			*NA*			*NI*		
	good									

Note: *OR,* odds ratio; *CI* confidence interval; *NI*, not included in the final logistic regression analysis; *NA*, not applicable.

Supplementary table 3. Binary logistic regression analysis of maternal knowledge, attitudes and practices related to neonatal jaundice for mothers without yuesao (N=265)

Variables	Classification	Knowledge			Attitude			Practices		
		OR	95%*CI*	*P*	*OR*	95%*CI*	*P*	*OR*	95%*CI*	*P*
Education level	High school and below (Ref.)			**0.001**	*NI*			*NI*		
	College and undergraduate course	2.832	1.430~5.610	0.030						
	Postgraduate student or above	20.140	3.036~133.601	0.002						
Average family monthly income (RMB)	≤5000 (Ref.)	*NI*			*NI*			*NI*		
	5001~10000									
	10001~20000									
	20001~30000									

For peer review only

	≥30001									
Prior education on neonatal jaundice	Yes (Ref.)					*NI*			*NI*	
	No	5.335	1.654~17.208	**0.005**						
Neonate sex	Female (Ref.)					*NI*			*NI*	
	Male	2.083	1.233~3.518	**0.006**						
Occupation	Employed (Ref.)		*NI*			*NI*			*NI*	
	Self-employed									
	Homemaker									
	Other									
Knowledge level	Poor (Ref.)		*NA*							
	good				1.734	1.059~2.847	**0.029**	3.012	1.757~5.165	**0.000**
Attitude level	poor (Ref.)		*NI*			*NA*				
	good							1.967	1.161~3.333	**0.012**

Note: *OR,* odds ratio; *CI* confidence interval; *NI*, not included in the final logistic regression analysis; *NA*, not applicable.

For peer review only

STROBE Statement—Checklist of items that should be included in reports of ***cross-sectional studies***

	Item No	Recommendation	Reported on page number
**Title and abstract**	1	(*a*) Indicate the study's design with a commonly used term in the title or the abstract	Page 1:title page
		(*b*) Provide in the abstract an informative and balanced summary of what was done and what was found	Page 2-3
**Introduction**			
Background/rationale	2	Explain the scientific background and rationale for the investigation being reported	Page 3-4
Objectives	3	State specific objectives, including any prespecified hypotheses	Page 4
**Methods**			
Study design	4	Present key elements of study design early in the paper	Page 4
Setting	5	Describe the setting, locations, and relevant dates, including periods of recruitment, exposure, follow-up, and data collection	Page 4 and Supplementary figure 1data collection progress
Participants	6	(*a*) Give the eligibility criteria, and the sources and methods of selection of participants	Page 4
Variables	7	Clearly define all outcomes, exposures, predictors, potential confounders, and effect modifiers. Give diagnostic criteria, if applicable	Page 6:The levels of knowledge, attitude and practice (for poor, for good)
Data sources/ measurement	8*	For each variable of interest, give sources of data and details of methods of assessment (measurement). Describe comparability of assessment methods if there is more than one group	Page 4-6
Bias	9	Describe any efforts to address potential sources of bias	Page 2-3 Strengths and limitations of this study
Study size	10	Explain how the study size was arrived at	NI:Sufficient sample size
Quantitative variables	11	Explain how quantitative variables were handled in the analyses. If applicable, describe which groupings were chosen and why	Page 6
Statistical methods	12	(*a*) Describe all statistical methods, including those used to control for confounding	Page 6
		(*b*) Describe any methods used to examine subgroups and interactions	Page 6
		(*c*) Explain how missing data were addressed	Page 5-6 excluded 10 participants' data

For peer review only

		(*d*) If applicable, describe analytical methods taking account of sampling strategy	NA
		(*e*) Describe any sensitivity analyses	NA
**Results**			
Participants	13*	(a) Report numbers of individuals at each stage of study—eg numbers potentially eligible, examined for eligibility, confirmed eligible, included in the study, completing follow-up, and analysed	Supplementary figure 1data collection progres
		(b) Give reasons for non-participation at each stage	Supplementary figure 1data collection progres
		(c) Consider use of a flow diagram	Supplementary figure 1data collection progres
Descriptive data	14*	(a) Give characteristics of study participants (eg demographic, clinical, social) and information on exposures and potential confounders	Page 7 and Table1
		(b) Indicate number of participants with missing data for each variable of interest	NI
Outcome data	15*	Report numbers of outcome events or summary measures	Page 6-7
Main results	16	(*a*) Give unadjusted estimates and, if applicable, confounder-adjusted estimates and their precision (eg, 95% confidence interval). Make clear which confounders were adjusted for and why they were included	Page 12 and Table5
		(*b*) Report category boundaries when continuous variables were categorized	NI
		(*c*) If relevant, consider translating estimates of relative risk into absolute risk for a meaningful time period	NA
Other analyses	17	Report other analyses done—eg analyses of subgroups and interactions, and sensitivity analyses	NA
**Discussion**			
Key results	18	Summarise key results with reference to study objectives	Page 15-17
Limitations	19	Discuss limitations of the study, taking into account sources of potential bias or imprecision. Discuss both direction and magnitude of any potential bias	Page 18
Interpretation	20	Give a cautious overall interpretation of results considering objectives, limitations, multiplicity of analyses, results from similar studies, and other relevant evidence	Page 18
Generalisability	21	Discuss the generalisability (external validity) of the study results	Page 18
**Other information**			
Funding	22	Give the source of funding and the role of the funders for the present study and, if applicable, for the original study on which the present article is based	Page 18-19

Note: *NI*, Not included ; *NA*, Not available

For peer review only - http://bmjopen.bmj.com/site/about/guidelines.xhtml
